# Genome-wide association studies identify polygenic effects for completed suicide in the Japanese population

**DOI:** 10.1038/s41386-019-0506-5

**Published:** 2019-09-02

**Authors:** Ikuo Otsuka, Masato Akiyama, Osamu Shirakawa, Satoshi Okazaki, Yukihide Momozawa, Yoichiro Kamatani, Takeshi Izumi, Shusuke Numata, Motonori Takahashi, Shuken Boku, Ichiro Sora, Ken Yamamoto, Yasuhiro Ueno, Tatsushi Toda, Michiaki Kubo, Akitoyo Hishimoto

**Affiliations:** 1 Department of Psychiatry, Kobe University Graduate School of Medicine, Kobe, Japan; 2 Laboratory for Statistical Analysis, RIKEN Center for Integrative Medical Sciences, Yokohama, Japan; 3 Department of Ophthalmology, Graduate School of Medical Sciences, Kyushu University, Fukuoka, Japan; 4 Department of Neuropsychiatry, Kindai University Faculty of Medicine, Osaka, Japan; 5 Laboratory for Genotyping Development, RIKEN Center for Integrative Medical Sciences, Yokohama, Japan; 6 Laboratory of Complex Trait Genomics, Department of Computational Biology and Medical Sciences, Graduate School of Frontier Sciences, The University of Tokyo, Tokyo, Japan; 7 Department of Pharmacology, Pharmaceutical Sciences, Health Sciences University of Hokkaido, Hokkaido, Japan; 8 Department of Psychiatry, Graduate School of Biomedical Sciences, Tokushima University, Tokushima, Japan; 9 Division of Legal Medicine, Kobe University Graduate School of Medicine, Kobe, Japan; 10 Department of Medical Biochemistry, Kurume University School of Medicine, Kurume, Japan; 11 Department of Neurology, Graduate School of Medicine, University of Tokyo, Tokyo, Japan; 12 RIKEN Center for Integrative Medical Sciences, Yokohama, Japan

**Keywords:** Genetic markers, Predictive markers

## Abstract

Suicide is a significant public health problem worldwide, and several Asian countries including Japan have relatively high suicide rates on a world scale. Twin, family, and adoption studies have suggested high heritability for suicide, but genetics lags behind due to difficulty in obtaining samples from individuals who died by suicide, especially in non-European populations. In this study, we carried out genome-wide association studies combining two independent datasets totaling 746 suicides and 14,049 non-suicide controls in the Japanese population. Although we identified no genome-wide significant single-nucleotide polymorphisms (SNPs), we demonstrated significant SNP-based heritability (35–48%; *P* < 0.001) for completed suicide by genomic restricted maximum-likelihood analysis and a shared genetic risk between two datasets (*P*_best_ = 2.7 × 10^−13^) by polygenic risk score analysis. This study is the first genome-wide association study for suicidal behavior in an East Asian population, and our results provided the evidence of polygenic architecture underlying completed suicide.

## Introduction

Suicide is a significant public health problem that causes nearly one million deaths worldwide each year [1]. In addition, several Asian countries including Japan have relatively high suicide rates on a world scale [2, 3].

There is evidence for high heritability in suicidal behavior, with an estimated *h*^2^ of 30–50% based on twin, family, and adoption studies in European populations [4]. To investigate the genetic components of suicidal behavior, large-scale genetic studies focusing on “completed suicide” should be crucial because other suicidal behaviors (suicide ideation or attempt) vary in terms of degree of lethality and suicidal intent, which may cause heterogeneity [5]. However, due to the difficulty in obtaining samples from individuals who died by suicide, few genome-wide association studies (GWASs) have been reported (only European populations) [6–9]. The largest case–control GWAS to date included 317 suicides, and revealed no significant findings [7]. In addition, no study has investigated single-nucleotide polymorphism (SNP)-based heritability or polygenic effects for completed suicide. Consequently, genetic insights into suicide lag behind those of other mental problems, and no useful genetic biomarker of suicide risk has been found.

On the other hand, previous studies have associated different characteristics with suicidal behavior in different age groups; for instance, more impulsive, aggressive, and novelty seeking personality traits in the young, while heightened harm avoidance and higher comorbidity rate of depression and physical diseases were associated with increasing age of suicidal behavior [10–13]. Family studies have indicated strong familial transmission for early-onset suicidal behavior [14–16]. These studies encourage research focusing on the biological factors associated with age at suicide.

Here, we first conducted genome-wide association analyses using two independent datasets totaling ~746 suicides and 14,049 non-suicide controls in the Japanese population in order to identify genetic variants affecting suicide risk (case–control study). We then utilized these genome-wide SNP data to investigate SNP-based heritability and polygenic effects for completed suicide. Additionally, we also investigated individual variants and polygenicity affecting age at suicide (targeting only suicidal cohorts).

## Materials and methods

### Subjects

The entire study design and procedures were performed in accordance with the Declaration of Helsinki. This study was approved by the Ethics Committee for Genetic Studies of Kobe University and RIKEN.

### Individuals who died by suicide

Autopsies on suicide victims were conducted at the Division of Legal Medicine in the Department of Community Medicine and Social Health Science at the Kobe University Graduate School of Medicine. The verdict of “completed suicide” was made through discussion with the Medical Examiner’s Office of the Hyogo Prefecture and the Division of Legal Medicine in the Kobe University Graduate School of Medicine [17]. In order to gather background information on completed suicides, psychological autopsy through their medical records and bereaved family interviews were conducted by professional staff from the Medical Examiner’s Office of the Hyogo Prefecture and the Division of Legal Medicine in the Kobe University, where available.

### Non-suicide controls

As non-suicide controls, we used genome-wide genotype data from subjects in the Biobank Japan project who had been genotyped as case subjects for non-psychiatric disorders and from healthy volunteers of the Osaka-Midosuji Rotary Club and the Pharma SNP consortium. The controls were not psychiatrically evaluated [18–20].

### Genotyping, QC, and imputation

We genotyped 434 individuals who died by suicide between June 1996 and July 2012 and 405 individuals who died by suicide between August 2012 and February 2017 using Illumina HumanOmniExpress and HumanOmniExpressExome BeadChips for the first and second set, respectively. We obtained control data genotyped with the same arrays (*N* = 7993 and 7136 for the first and second set, respectively) (Table S1).

We performed quality control (QC) using PLINK 1.9 [21]. First, we excluded SNPs with a call rate <0.98 and minor allele frequency (MAF) <0.01, and those with *P* < 1.0 × 10^−6^ for Hardy–Weinberg equilibrium (HWE) in controls. Related individuals were excluded (PI_HAT ≥0.175). We then performed principal component analysis (PCA), and excluded samples outside the Japanese main islands cluster [22, 23]. The results of PCA are shown in Fig. S1. The final datasets included 386 suicides and 7458 controls as the first set, and 360 suicides and 6591 controls as the second set. After estimating haplotypes using SHAPEIT2 (v2.r778) [24], we performed genotype imputation by Minimac3 (1.0.13) [25] using ALL samples in the 1000 Genomes Project phase 3v5 [26] as a reference.

To investigate the X chromosome, we called genotypes using GenomeStudio. First, we generated genoplots using only female samples. After that, we added male samples to genoplots and called genotypes. Genotypes called as heterozygotes were treated as missing in male samples. For the genotyping QC, we excluded SNPs with MAF <0.01 and SNP call rate <0.98 in either male or female samples. We also excluded SNPs with *P* value for HWE <1.0 × 10^−6^ in female samples. Haplotype phasing and imputation were performed separately for males and females. Allelic dosages were imputed from 0 to 2 in male samples under the assumption of full dosage compensation. The pseudo-autosomal region was excluded from the reference before imputation.

After imputation of autosomal and X chromosomes, we included only SNPs with high imputation quality (*r*^2^ ≥ 0.7) and MAF ≥0.01 (Fig. S2).

### Association analysis and meta-analysis

For all GWASs identifying genetic variants affecting suicide risk (case–control study; 746 suicides and 14,049 controls) and age at suicide (only suicidal cohorts; 719 suicides), single variant association tests were performed for common variants (MAF ≥0.01) using logistic regression and linear regression based on Wald test implemented in the Rvtests software [27] with a correction for the top 10 PCs as covariates. Meta-analysis was performed with the METAL software [28] using a fixed-effects model with inverse-variance weighted approach. The significance level was set at *P* < 2.5 × 10^−8^ due to correction for multiple comparison for two GWASs (case–control GWAS and GWAS for age at suicide). *P* for heterogeneity between two analyses (first set and second set) was calculated by Cochran’s *Q* test. Regional association plots were generated using LocusZoom [29]. A QQ plot for each GWAS is shown in Fig. S3.

### Evaluation of previously implicated SNPs in prior GWAS of suicidal behavior

Among variants that have previously been associated with suicidal behavior (suicide ideation, suicide attempt, and suicide completion) in the published literature [7, 9, 30–36], we identified 26 variants with *P* < 1.0 × 10^−6^ in previous GWASs and MAF >0.01 in JPT (Japanese in Tokyo, Japan) population (the 1000 Genomes Project phase 3). We looked up the association of each of these SNPs with the results from our case–control GWAS. Only the top SNP in the same region in each reference was selected. For a candidate SNP that was not directly genotyped or imputed in our GWAS, we identified a proxy SNP (*r*^2^ > 0.8 in East Asian samples of the 1000 Genomes Project phase 3) whenever possible. For SNPs that reached *P* < 0.05, we determined whether the direction of the association was consistent between the prior and current studies.

### Estimation of the proportion of the variance in completed suicide explained by the genotyped SNPs (SNP-based heritability)

To assess SNP-based heritability (*h*^2^_SNP_), we used genome-wide complex trait analysis (GCTA) [37] to generate genetic relatedness matrices (GRMs) among 506,645 SNPs that passed QC in both genotyped datasets, and then performed genomic restricted maximum-likelihood (GREML) analysis. This assumed prevalence rates of 0.1 and 0.5% for completed suicide, considering the reported incidence of completed suicide in Japan (Ministry of Health, Labor, and Welfare of Japan) [3] and the estimates from previous papers [38–40]. We strictly controlled the cryptic relatedness of the analyzed samples using the –grm-cutoff option (threshold of 0.05) implemented by GCTA, including 385 cases and 7409 controls for the first set, and 357 cases and 6560 controls for the second set, respectively. We then estimated SNP-based heritability using reml function implemented by GCTA with top 10 PCs as covariates.

### PRS analysis

Polygenic risk score (PRS) analyses were performed using PRSice v1.23 [41]. The *P* threshold (*P*_t_) for selecting “risk” SNPs was sequentially set at 0.1, 0.2, 0.3, 0.4, and 0.5 without SNPs in the major histocompatibility complex region. We then performed linkage disequilibrium (LD) clumping (used by the default setting of the software) to select the eligible SNPs for PRS. To calculate the PRS, we analyzed two discovery/target sets, using the first set (386 suicides and 7458 controls) as the discovery set and the second set (360 suicides and 6591 controls) as the target set, and vice versa. We included the top 10 PCs derived from each genotyped dataset as covariates, respectively. The variance explained for the PRS was estimated based on Nagelkerke’s *R*^2^ from a logistic regression model. For the analysis of polygenic effects on age at suicide (first set, 366 suicides; second set, 353 suicides), we also applied the same procedure as the above to the case–control GWAS datasets.

### Pathway enrichment analysis

Using the results of a meta-analysis of the case–control GWAS datasets, we ran PASCAL [42] for gene-based enrichment analysis using 1077 gene sets, including KEGG [43], REACTOME [44], and BIOCARTA (http://cgap.nci.nih.gov/Pathways/BioCarta_Pathways). The significance level was set at *χ*^2^ *P* < 4.6 × 10^−5^ after Bonferroni correction for 1077 tests.

## RESULTS

### GWAS for risk of completed suicide

Sample characteristics are shown in Table S1. We performed a meta-analysis of the case–control GWAS datasets for both suicides and non-suicide controls (totaling 746 cases and 14,049 controls) using 8,625,325 SNPs (λ genomic control (GC) = 1.07; Fig. S3a). This analysis identified no genome-wide significant SNPs (Fig. S4), although some loci, including SNPs on *GRM1* and *CTPS2*, were suggestive with *P*_meta_ < 1.0 × 10^−6^ (Table S2). In addition, we looked up the 26 variants showing *P* < 1.0 × 10^−6^ in prior European GWASs for suicidal behavior [7, 9, 30–36]. Among these, rs7989250, reported in the recent UK Biobank GWAS for ordinal suicidality, reached the threshold for our replication analysis (*P* < 0.05) with the same direction of allelic effect (Table S3). We ran PASCAL [42] using our meta-GWAS to estimate the enrichments in 1077 gene sets, but no pathways were enriched at the Bonferroni level of significance (Results for all tested 1077 pathways are listed in an Excel spreadsheet that is included in the Supplementary Material).

### GREML analysis reveals high SNP-based heritability for completed suicide

**Fig. 1:**
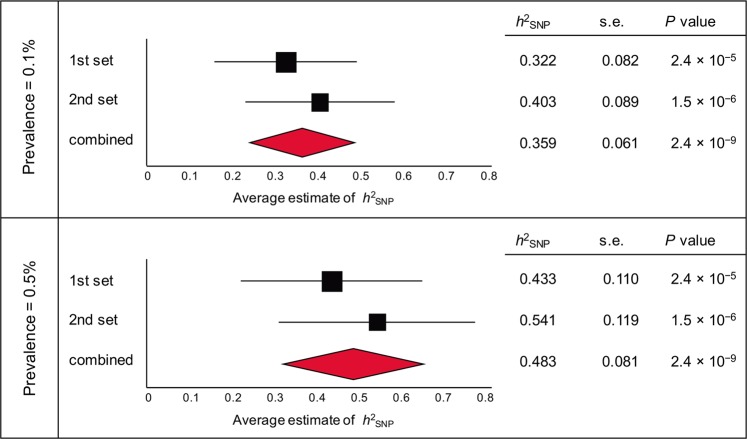
Forest plot showing estimated SNP-based heritability in completed suicide. SNP-based heritability (*h*^2^_SNP_) was estimated with assumed prevalence rates of 0.1 and 0.5% using the GREML method. Averages and 95% confidence intervals are shown

To investigate SNP-based heritability (*h*^2^_SNP_) for completed suicide, we used GCTA [37] to GRMs using 506,645 directly genotyped SNPs, and then estimated SNP-based heritability with GREML. These analyses revealed significant SNP-based heritability in both datasets (*P* < 0.001; Fig. 1). By combining the results, we estimated that 35.9% and 48.3% of phenotypic variance in the two datasets could be explained by SNPs with a prevalence of 0.1% and 0.5%, respectively; this implies the underlying polygenic architecture composed by numerous “risk” SNPs for completed suicide.

### PRS analysis reveals polygenic effects for completed suicide

**Fig. 2:**
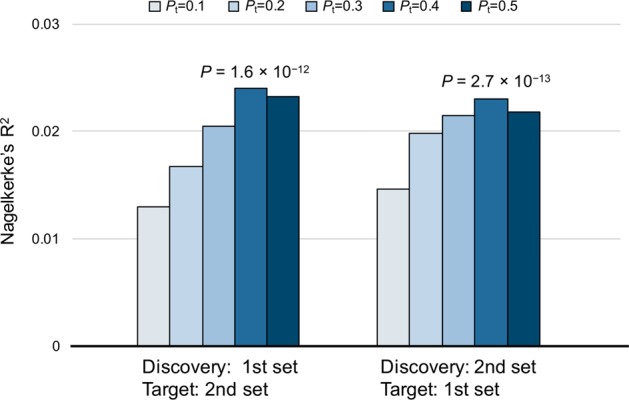
Polygenic risk score (PRS) analysis of completed suicide. We analyzed two discovery/target sets, using the first set as the discovery set and the second set as the target set, and vice versa. The *P* threshold (*P*_t_) for selecting “risk” SNPs was sequentially set at 0.1, 0.2, 0.3, 0.4, and 0.5 without SNPs in the major histocompatibility complex (MHC) region. The *y*-axis indicates the explained variation (Nagelkerke’s *R*^2^)

To investigate whether two independent datasets share genetic components conferring the risk of completed suicide, we performed PRS analyses by PRSice (v1.23) [41], setting the first set as the discovery set and the second set as the target set, and vice versa. The analyses demonstrated significant polygenic effects on completed suicide (*P* < 0.001) when the SNPs were stratified by *P* values, obtaining the most significant *P* values (*P*_best_ = 2.7 × 10^−13^) with an inclusion threshold of *P*_t_ = 0.4 in each set, with explained 1.3–2.4% of the variance (Fig. 2 and Table S4). These results strongly suggest shared genetic components for completed suicide between the two datasets, providing additional evidence of polygenic architecture underlying completed suicide.

### GWAS for age at suicide

**Fig. 3:**
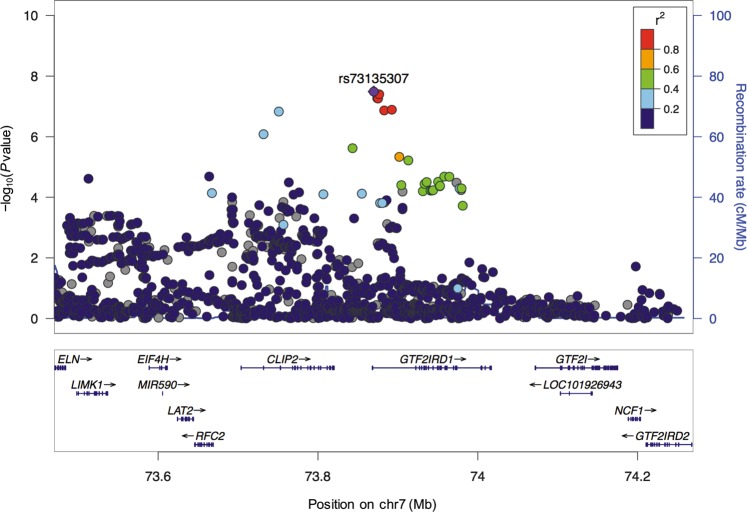
Regional plots of the top hit in the association results based on the meta-analysis of the first and second sets. Blue lines indicate the recombination rate for East Asian samples of the 1000 Genomes Project phase 3. The *y*-axis is −log_10_(*P* value) of the SNPs and the *x-*axis is chromosomal position (hg19). The linkage disequilibrium (*r*^2^) between the top and the remaining SNPs is indicated by color

Age-divided sample characteristics focusing on psychological background information, comorbid severe physical diseases, and distribution of age at suicide in both the first and second set of our suicidal cohorts are shown in Table S5 and Fig. S5. We performed GWAS for the age at completed suicide in each dataset (only suicides with accurate age information available; *N* = 366 and 353 for the first and second set, respectively), and integrated these results by fixed-effects meta-analysis. The meta-GWAS included 8,810,873 SNPs, and the genomic inflation factor suggested low possibility of bias from population stratification and cryptic relatedness (λ GC = 1.02; a QQ plot is shown in Fig. S3b). We identified a novel suggestive locus on 7q11.23 in GTF2I repeat domain containing 1 (*GTF2IRD1*) (top SNP rs73135307 G > C; *P* = 3.3 × 10^−8^, *β* = −12.3 years; Fig. 3, Table 1, Table S6, and Fig. S6) without significant heterogeneity between the two datasets (*P*_het_ = 0.20). This means that the effect allele of rs73135307 (G allele) could lead to 12.3 years younger of age at suicide in our suicidal cohort compared to C allele. G allele frequency variation of rs73135307 in divided groups based on age at suicide in the first and second set are shown in Fig. S7. According to HaploReg (v4.1) [45], rs73135307 and the four variants in LD (*r*^2^ > 0.6 in East Asian samples of the 1000 Genomes Project [26]) with rs73135307 affect various histone modifications (H3K4me1, H3K4me3, H3K27ac, and H3K9ac) in various brain regions, including the hippocampus and dorsolateral prefrontal cortex. However, neither nonsynonymous SNP nor significant expression quantitative trait loci (eQTL) was found. Our PRS analysis showed no significant polygenic effects on age at suicide (Table S7).

**Table 1:** Top variant identified by meta-analysis of genome-wide association analyses of age at completed suicide

SNP	Chr^a^	Position^a^ (bp)	Gene^a^ (location)	Effect allele (REF allele)	Stage	*β* ^b^	s.e.	*P* value	*P*_het_^c^
rs73135307	7	73,869,857	*GTF2IRD1* (intronic)	G (C)	First set	−9.84	2.97	9.17 × 10^−4^	
					Second set	−15.6	3.39	4.15 × 10^−6^	
					Meta-analysis	−12.3	2.23	**3.25** **×** **10**^−**8**^	0.20

Suggestive significance (*P* < 5.0 × 10^−8^) after meta-analysis of first and second set is shown in bold. Only top SNP in the same region is shown here. Full results (*P* < 1.0 × 10^−6^) can be seen in Table S6

*Chr* chromosome, *bp* base pair, *REF* reference allele

^a^Position and gene are based on Human Genome version 19 (hg19), build 37

^b^*β* were calculated by linear regression based on Wald tests implemented in Rvtests software

^c^*P* for heterogeneity between two analyses (first set and second set) were calculated by Cochran’s *Q* test

## Discussion

Through the GWAS including 746 suicides in the Japanese population, we demonstrated SNP-based heritability (35–48%) and polygenic effects, indicating polygenic inheritance of completed suicide. We also showed that the *GTF2IRD1* locus is suggestively associated with age at completed suicide.

Using the two case–control GWAS datasets, we estimated SNP-based heritability with GREML and conducted PRS analyses. Our GREML analysis revealed that completed suicide has significant SNP-based heritability, with estimates of 35–48% in both individual GWAS sets. We estimated SNP-based heritability using the GREML method with two different prevalence rates (0.1 and 0.5%) assumed from the reported incidence of completed suicide in Japan [3] and the previous studies [38–40]. These estimates are qualified by the difficulty of estimating the lifetime prevalence of completed suicide, compared to those of “diseases,” presumably due to the influence of historical and cultural contexts on the epidemiology of suicide, along with other national/regional differences [46]. Moreover, our PRS analyses demonstrated significant polygenic effects on completed suicide, indicating that two independent cohorts of suicides shared genetic components conferring suicide risk. Consequently, to our knowledge, we provide the first evidence of SNP-based heritability in completed suicide derived from case–control GWAS dataset, other than from epidemiological data such as twin, family, and adoption studies.

Due to the lack of well-designed epidemiological studies and GWAS for suicidal behavior in Asia, this is the first study demonstrating SNP-based heritability of suicide in an Asian population. This is a major strength of the current study, since the dearth of GWASs in non-European populations compared with the abundance of European-descent studies is causing the disparity and poor generalizability of genetic studies across populations [47]. The SNP-based heritability of completed suicide found in our analysis dramatically exceeded the previous estimates from past GWASs of suicide attempt in European populations, and further comparable to twin heritability estimated by prior studies of European populations despite SNP-based heritability estimates by GWASs usually explaining roughly half of those based on twin modeling [48]. In order to elucidate the reasons for the different levels of SNP-based heritability estimated by previous GWASs for suicide attempt and our GWAS, we should focus on the following points regarding SNP-based heritability differences: (1) between suicide attempt and suicide completion and (2) between Europeans and Asians. On the other hand, significant high SNP-based heritability for completed suicide shown here might be overestimated to some extent due to residual population stratification (e.g., regional differences in Japan) or the genetic effects of comorbid psychiatric disorders. Indeed, genetic influences on suicidal behavior seem to be confounded by genetic vulnerability for psychiatric disorders; for instance, a previous twin study reported lower heritability (~17%) for suicidal behavior after controlling for the inheritance of psychiatric disorders [49]. Publicly available GWAS data focusing on psychiatric problems in East Asian population are limited, which makes difficult to perform replication of GWAS for suicidal behavior and in-depth analyses (e.g., suicide GWAS divided into cases with and without psychiatric disorders, or genetic correlation analysis with various psychiatric disorders/conditions) using GWAS datasets from the same Asian population. Since we have made our summary statistics of GWAS publicly available, our data will enable the above analyses in the future.

Our meta-analysis of two GWASs of age at completed suicide identified a suggestive locus, intronic SNPs in the *GTF2IRD1* gene (top hit rs73135307, *P* = 3.3 × 10^−8^) at 7q11.23. This SNP and variants in LD with the lead SNP are associated with various histone modifications across brain regions related to suicide pathophysiology [5], although none of those SNPs were nonsynonymous or overlapped with eQTLs. *GTF2IRD1* has been reported as one of the promising genes for Williams syndrome (WS), particularly responsible for neurodevelopmental abnormalities [50], which is known as a risk factor for suicidal behavior [51]. While the typical social phenotype of WS is characterized by optimistic personality, there is also evidence of diametric characteristics in individuals with WS, such as heightened anxiety, social relationship difficulties, and higher prevalence of autism spectrum disorder (ASD) [52–54]. The associations of *GTF2IRD1* with anxiety and social impairment in WS have been previously reported [53, 55]. In addition, various copy number variations in 7q11.23 region have been strikingly implicated in the genetic etiology of schizophrenia and ASD [56, 57], which are associated with high risk of youth suicide [58, 59]. Focusing on this chromosomal region by more detailed genome sequencing is necessary to yield further insight into the genetic feature of suicide in young people. Indeed, the aberrant personality traits, lower comorbidity rate of depression and physical diseases, and familial transmission previously linked with younger age at suicidal behavior [10–16] indicated the possibility that future genetic studies for suicide in young people may identify genetic clues.

It must be emphasized that our findings should be interpreted in the context of several other limitations. First, although our sample size was one of the largest ever for research on completed suicide, larger sample sizes would be preferable in order to draw robust conclusions regarding SNP-based heritability and polygenicity for completed suicide, and to detect reliable genetic markers. In particular, although we reported that the *GTF2IRD1* locus is suggestively associated with age at completed suicide, GWAS for age at suicide here contained too small sample size (*N* = 719) and replication studies are indeed required. Second, our study cohort was restricted to only the Japanese population; thus, findings from the present study might not be generalizable to other populations. Third, the subjects we used non-suicide controls who had not been psychiatrically screened. In addition, most of the controls had various non-psychiatric disorders [18–20]. However, this approach has been already applied in the previous GWAS [20] of which results was replicable in other recent GWAS [60], supporting the reliability of our results. Fourth, the biological interpretation of the significant polygenic effects for completed suicide and the variants reported here remains largely unknown due to lack of functional evidence yielded by our pathway analysis and database searches, respectively.

In conclusion, we provided the first evidence of SNP-based heritability and polygenic effects in completed suicide, and polygenic effects in age at suicide.

## Funding and disclosure

This work was supported, in part, by JSPS KAKENHI Grant Number JP17H04249, BBJ, and the Rotary Club of Osaka-Midosuji District 2660 Rotary International in Japan. The authors declare no competing interests.

## Supplementary information

Supplementary Figures

Supplementary Tables

Supplementary Information

## References

[1] World Health Organization. Suicide fact sheet; 2017. http://www.who.int/mediacentre/factsheets/fs398/en/. Accessed 29 Aug 2017.

[2] Värnik P (2012). Suicide in the world. Int J Environ Res Public Health.

[3] Statistics and Information Department, Minister’s Secretariat, Ministry of Health, Labour and Welfare. Vital statistics of Japan; 2017. https://www.mhlw.go.jp/wp/hakusyo/jisatsu/19/index.html.

[4] Brent DA, Turecki G (2017). Suicide and suicidal behavior. Lancet.

[5] Oquendo MA, Sullivan GM, Sudol K, Baca-Garcia E, Stanley BH, Sublette E (2014). Toward a biosignature for suicide. Am J Psychiatry.

[6] Coon H, Darlington TM, DiBlasi E, Callor WB, Ferris E, Fraser A, et al. Genome-wide significant regions in 43 Utah high-risk families implicate multiple genes involved in risk for completed suicide. Mol Psychiatry. 2018. https://www.nature.com/articles/s41380-018-0282-3. [Epub ahead of print]. 10.1038/s41380-018-0282-3 PMC6478563 30353169

[7] Galfalvy H, Haghighi F, Hodgkinson C, Goldman D, Oquendo MA, Burke A (2015). A genome-wide association study of suicidal behavior. Am J Med Genet B.

[8] Mirkovic B, Laurent C, Podlipski MA, Frebourg T, Cohen D, Gerardin P (2016). Genetic association studies of suicidal behavior: a review of the past 10 years, progress, limitations, and future directions. Front Psychiatry.

[9] Strawbridge RJ, Ward J, Ferguson A, Graham N, Shaw RJ.Cullen B, et al. Identification of novel genome-wide associations for suicidality in UK Biobank, genetic correlation with psychiatric disorders and polygenic association with completed suicide. EBioMedicine. 2019;41:517–25. 10.1016/j.ebiom.2019.02.005 PMC6442001 30745170

[10] Bozzay ML, Liu RT, Kleiman EM (2014). Gender and age differences in suicide mortality in the context of violent death: findings from a multi-state population-based surveillance system. Compr Psychiatry.

[11] Fässberg MM, Cheung G, Canetto SS, Erlangsen A, Lapierre S, Lindner R (2016). A systematic review of physical illness, functional disability, and suicidal behaviour among older adults. Aging Ment Health.

[12] McGirr A, Renaud J, Bureau A, Seguin M, Lesage A, Turecki G (2008). Impulsive-aggressive behaviors and completed suicide across the life cycle: a predisposition for younger age of suicide. Psychol Med.

[13] Rich CL, Young D, Fowler RC (1986). San Diego suicide study. I. Young vs old subjects. Arch Gen Psychiatry.

[14] Brent DA, Melhem NM, Oquendo M, Burke A, Birmaher B, Stanley B (2015). Familial pathways to early-onset suicide attempt: a 5.6-year prospective study. JAMA Psychiatry.

[15] Brent DA, Oquendo M, Birmaher B, Greenhill L, Kolko D, Stanley B (2002). Familial pathways to early-onset suicide attempt: risk for suicidal behavior in offspring of mood-disordered suicide attempters. Arch Gen Psychiatry.

[16] Melhem N, Brent DA, Ziegler M, Iyengar S, Kolko D, Oquendo M (2007). Familial pathways to early-onset suicidal behavior: familial and individual antecedents of suicidal behavior. Am J Psychiatry.

[17] Otsuka I, Izumi T, Boku S, Kimura A, Zhang Y, Mouri K (2017). Aberrant telomere length and mitochondrial DNA copy number in suicide completers. Sci Rep.

[18] Nagai A, Hirata M, Kamatani Y, Muto K, Matsuda K, Kiyohara Y (2017). Overview of the BioBank Japan Project: study design and profile. J Epidemiol.

[19] Hirata M, Kamatani Y, Nagai A, Kiyohara Y, Ninomiya T, Tamakoshi A (2017). Cross-sectional analysis of BioBank Japan clinical data: a large cohort of 200,000 patients with 47 common diseases. J Epidemiol.

[20] Ikeda M, Takahashi A, Kamatani Y, Okahisa Y, Kunugi H, Mori N (2018). A genome-wide association study identifies two novel susceptibility loci and trans population polygenicity associated with bipolar disorder. Mol Psychiatry.

[21] Purcell S, Neale B, Todd-Brown K, Thomas L, Ferreira MA, Bender D (2007). PLINK: a tool set for whole-genome association and population-based linkage analyses. Am J Hum Genet.

[22] Price AL, Patterson NJ, Plenge RM, Weinblatt ME, Shadick NA, Reich D (2006). Principal components analysis corrects for stratification in genome-wide association studies. Nat Genet.

[23] Yamaguchi-Kabata Y, Nakazono K, Takahashi A, Saito S, Hosono N, Kubo M (2008). Japanese population structure, based on SNP genotypes from 7003 individuals compared to other ethnic groups: effects on population-based association studies. Am J Hum Genet.

[24] Delaneau O, Marchini J, Zagury JF (2011). A linear complexity phasing method for thousands of genomes. Nat Methods.

[25] Li Y, Willer CJ, Ding J, Scheet P, Abecasis GR (2010). MaCH: using sequence and genotype data to estimate haplotypes and unobserved genotypes. Genet Epidemiol.

[26] Abecasis GR, Altshuler D, Auton A, Brooks LD, Durbin RM, Gibbs RA (2010). A map of human genome variation from population-scale sequencing. Nature.

[27] Zhan X, Hu Y, Li B, Abecasis GR, Liu DJ (2016). RVTESTS: an efficient and comprehensive tool for rare variant association analysis using sequence data. Bioinformatics.

[28] Willer CJ, Li Y, Abecasis GR (2010). METAL: fast and efficient meta-analysis of genomewide association scans. Bioinformatics.

[29] Pruim RJ, Welch RP, Sanna S, Teslovich TM, Chines PS, Gliedt TP (2010). LocusZoom: regional visualization of genome-wide association scan results. Bioinformatics.

[30] Willour VL, Seifuddin F, Mahon PB, Jancic D, Pirooznia M, Steele J (2012). A genome-wide association study of attempted suicide. Mol Psychiatry.

[31] Erlangsen A, Appadurai V, Wang Y, Turecki G, Mors O, Werge T, et al. Genetics of suicide attempts in individuals with and without mental disorders: a population-based genome-wide association study. Mol Psychiatry. 2018. https://www.nature.com/articles/s41380-018-0218-y. [Epub ahead of print]. 10.1038/s41380-018-0218-y PMC7515833 30116032

[32] Kimbrel NA, Garrett ME, Dennis MF, Hauser MA, Ashley-Koch AE (2018). A genome-wide association study of suicide attempts and suicidal ideation in U.S. military veterans. Psychiatry Res.

[33] Levey DF, Polimanti R, Cheng Z, Zhou H, Nuñez YZ, Jain S (2019). Genetic associations with suicide attempt severity and genetic overlap with major depression. Transl Psychiatry.

[34] Mullins N, Perroud N, Uher R, Butler AW, Cohen-Woods S, Rivera M (2014). Genetic relationships between suicide attempts, suicidal ideation and major psychiatric disorders: a genome-wide association and polygenic scoring study. Am J Med Genet B.

[35] Perroud N, Uher R, Ng MY, Guipponi M, Hauser J, Henigsberg N (2012). Genome-wide association study of increasing suicidal ideation during antidepressant treatment in the GENDEP project. Pharmacogenomics J.

[36] Stein MB, Ware EB, Mitchell C, Chen CY, Borja S, Cai T (2017). Genomewide association studies of suicide attempts in US soldiers. Am J Med Genet B.

[37] Yang J, Lee SH, Goddard ME, Visscher PM (2011). GCTA: a tool for genome-wide complex trait analysis. Am J Hum Genet.

[38] Kessler RC, Borges G, Walters EE (1999). Prevalence of and risk factors for lifetime suicide attempts in the National Comorbidity Survey. Arch Gen Psychiatry.

[39] Bostwick JM, Pabbati C, Geske JR, McKean AJ (2016). Suicide attempt as a risk factor for completed suicide: even more lethal than we knew. Am J Psychiatry.

[40] Nock MK, Green JG, Hwang I, McLaughlin KA, Sampson NA, Zaslavsky AM (2013). Prevalence, correlates, and treatment of lifetime suicidal behavior among adolescents: results from the National Comorbidity Survey Replication Adolescent Supplement. JAMA Psychiatry.

[41] Euesden J, Lewis CM, O’Reilly PF (2015). PRSice: Polygenic Risk Score software. Bioinformatics.

[42] Lamparter D, Marbach D, Rueedi R, Kutalik Z, Bergmann S (2016). Fast and rigorous computation of gene and pathway scores from SNP-based summary statistics. PLoS Comput Biol.

[43] Kanehisa M, Goto S, Sato Y, Furumichi M, Tanabe M (2012). KEGG for integration and interpretation of large-scale molecular data sets. Nucleic Acids Res.

[44] Croft D, O’Kelly G, Wu G, Haw R, Gillespie M, Matthews L (2011). Reactome: a database of reactions, pathways and biological processes. Nucleic Acids Res.

[45] Ward LD, Kellis M (2012). HaploReg: a resource for exploring chromatin states, conservation, and regulatory motif alterations within sets of genetically linked variants. Nucleic Acids Res.

[46] Russell R, Metraux D, Tohen M (2017). Cultural influences on suicide in Japan. Psychiatry Clin Neurosci.

[47] Martin AR, Kanai M, Kamatani Y, Okada Y, Neale BM, Daly MJ (2019). Clinical use of current polygenic risk scores may exacerbate health disparities. Nat Genet.

[48] Yang J, Zeng J, Goddard ME, Wray NR, Visscher PM (2017). Concepts, estimation and interpretation of SNP-based heritability. Nat Genet.

[49] Fu Q, Heath AC, Bucholz KK, Nelson EC, Glowinski AL, Goldberg J (2002). A twin study of genetic and environmental influences on suicidality in men. Psychol Med.

[50] Fan CC, Schork AJ, Brown TT, Spencer BE, Akshoomoff N, Chen CH (2018). Williams syndrome neuroanatomical score associates with GTF2IRD1 in large-scale magnetic resonance imaging cohorts: a proof of concept for multivariate endophenotypes. Transl Psychiatry.

[51] Sokolowski M, Wasserman J, Wasserman D (2016). Polygenic associations of neurodevelopmental genes in suicide attempt. Mol Psychiatry.

[52] Klein-Tasman BP, Li-Barber KT, Magargee ET (2010). Honing in on the social phenotype in Williams syndrome using multiple measures and multiple raters. J Autism Dev Disord.

[53] Kopp ND, Parrish PCR, Lugo M, Dougherty JD, Kozel BA (2018). Exome sequencing of 85 Williams-Beuren syndrome cases rules out coding variation as a major contributor to remaining variance in social behavior. Mol Genet Genom Med.

[54] Richards C, Jones C, Groves L, Moss J, Oliver C (2015). Prevalence of autism spectrum disorder phenomenology in genetic disorders: a systematic review and meta-analysis. Lancet Psychiatry.

[55] Schneider T, Skitt Z, Liu Y, Deacon RM, Flint J, Karmiloff-Smith A (2012). Anxious, hypoactive phenotype combined with motor deficits in Gtf2ird1 null mouse model relevant to Williams syndrome. Behav Brain Res.

[56] Marshall CR, Howrigan DP, Merico D, Thiruvahindrapuram B, Wu W, Greer DS (2017). Contribution of copy number variants to schizophrenia from a genome-wide study of 41,321 subjects. Nat Genet.

[57] Sanders SJ, He X, Willsey AJ, Ercan-Sencicek AG, Samocha KE, Cicek AE (2015). Insights into autism spectrum disorder genomic architecture and biology from 71 risk loci. Neuron.

[58] Radomsky ED, Haas GL, Mann JJ, Sweeney JA (1999). Suicidal behavior in patients with schizophrenia and other psychotic disorders. Am J Psychiatry.

[59] Chen MH, Pan TL, Lan WH, Hsu JW, Huang KL, Su TP (2017). Risk of Suicide attempts among adolescents and young adults with autism spectrum disorder: a Nationwide Longitudinal Follow-Up Study. J Clin Psychiatry.

[60] Stahl EA, Breen G, Forstner AJ, McQuillin A, Ripke S, Trubetskoy V (2019). Genome-wide association study identifies 30 loci associated with bipolar disorder. Nat Genet.

