# Supplementary material for: Genome-wide association studies identify polygenic effects for completed suicide in the Japanese population
Source: Neuropsychopharmacology. 2019 Sep 2;44(12):2119–24. doi: 10.1038/s41386-019-0506-5 (PMC6887868; doi:10.1038/s41386-019-0506-5)
Supplement: Supplementary file 1 — Supplementary Figures [file 41386_2019_506_MOESM1_ESM.pdf]

Genome-wide association studies identify polygenic effects for completed suicide in the Japanese population

Supplementary Figures:

Figure S1. Results of principal component analysis (PCA)

Figure S2. Overview of the study

Figure S3. QQ plot of GWAS

Figure S4. Manhattan plot of case-control GWAS

Figure S5. Histogram of age at suicide

Figure S6. Manhattan plot of GWAS for age at suicide

Figure S7. G allele frequency variation of rs73135307 in divided groups based on age at suicide

a) PCA for case, control and the four populations (JPT, CHB, CEU, and YRI of the HapMap) in the 1st set

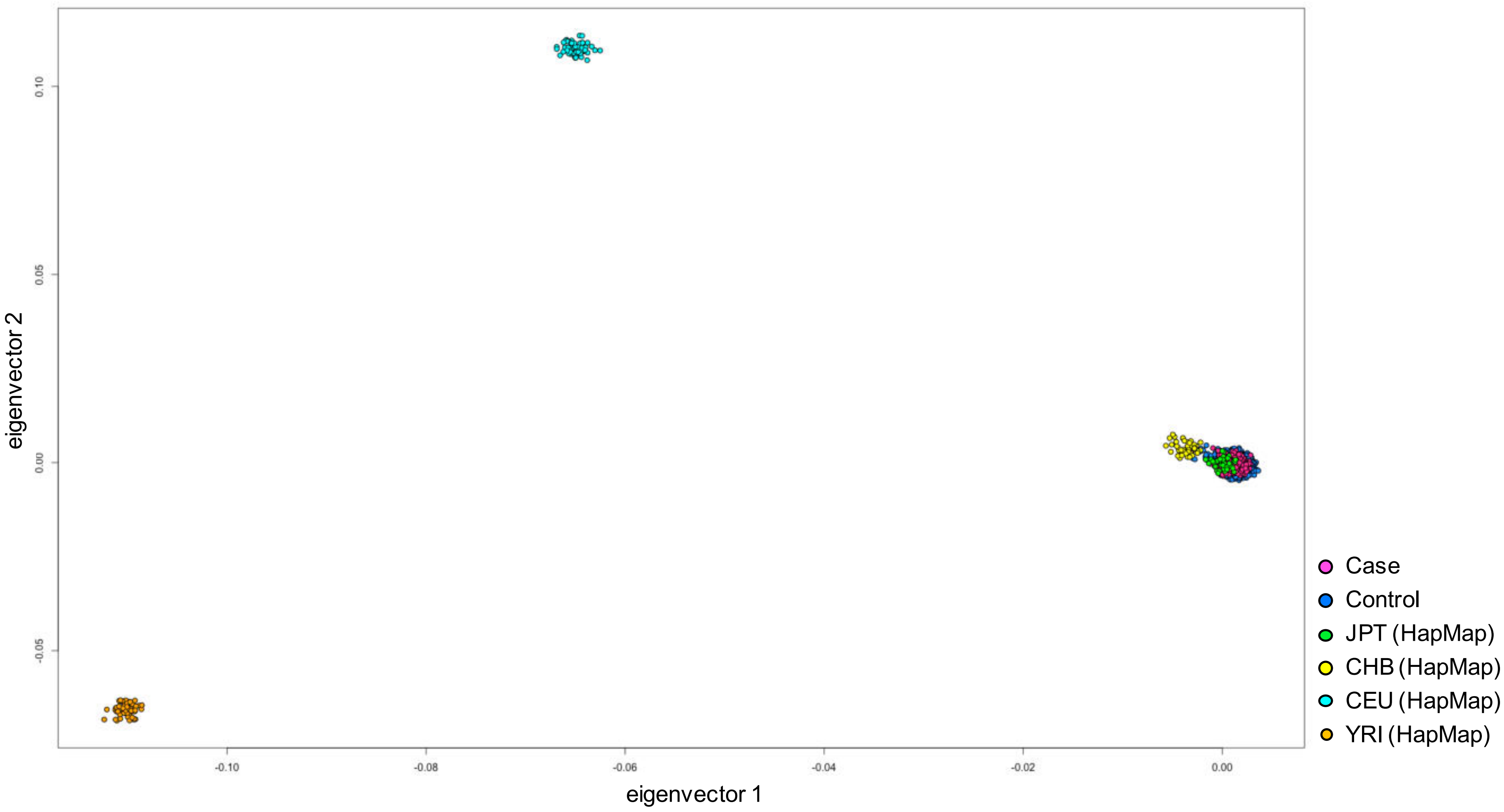

b) PCA for the East Asian population (case, control, and JPT and CHB of the HapMap) in the 1st set

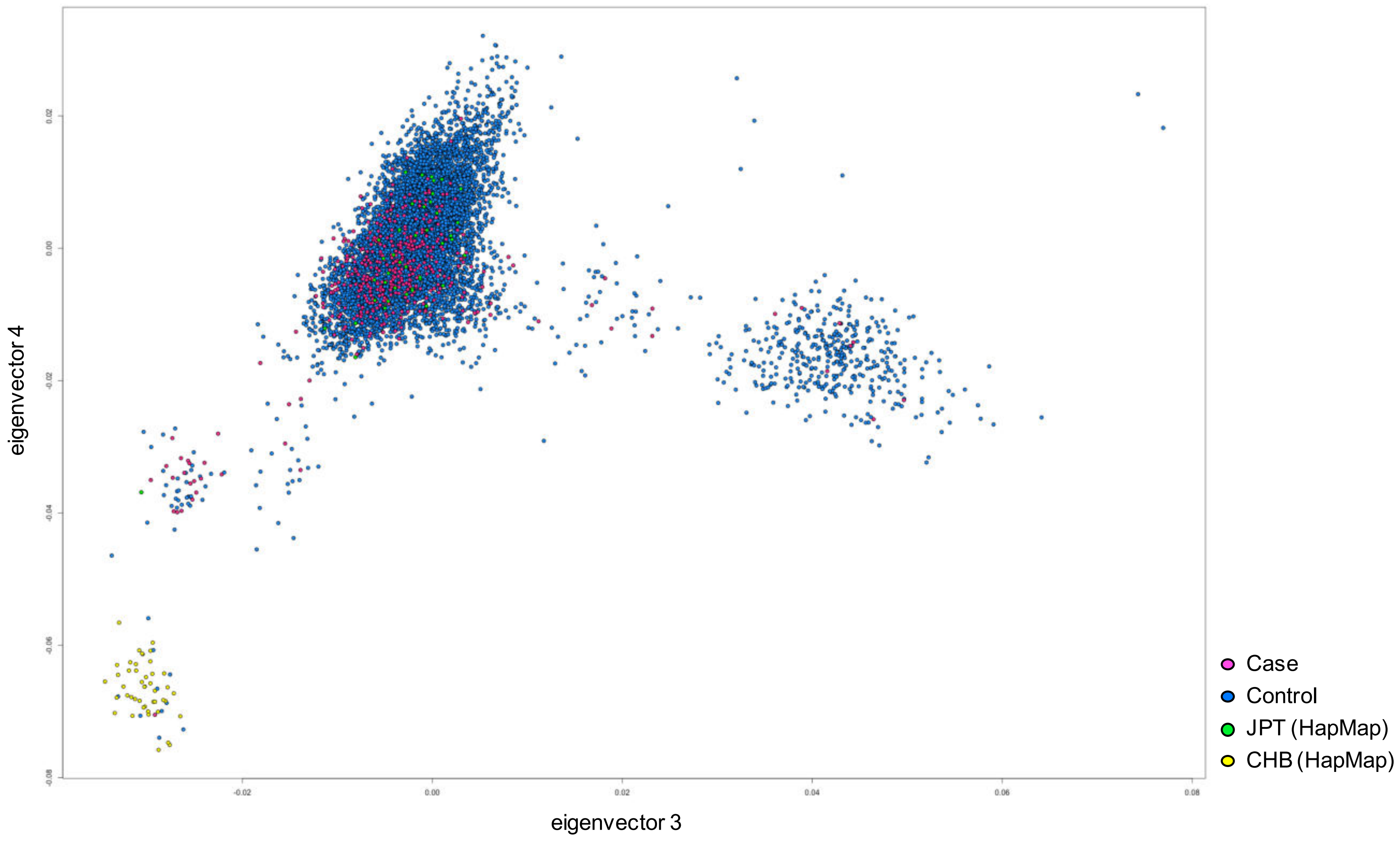

c) PCA for the Japanese cluster of the main islands in the 1st set

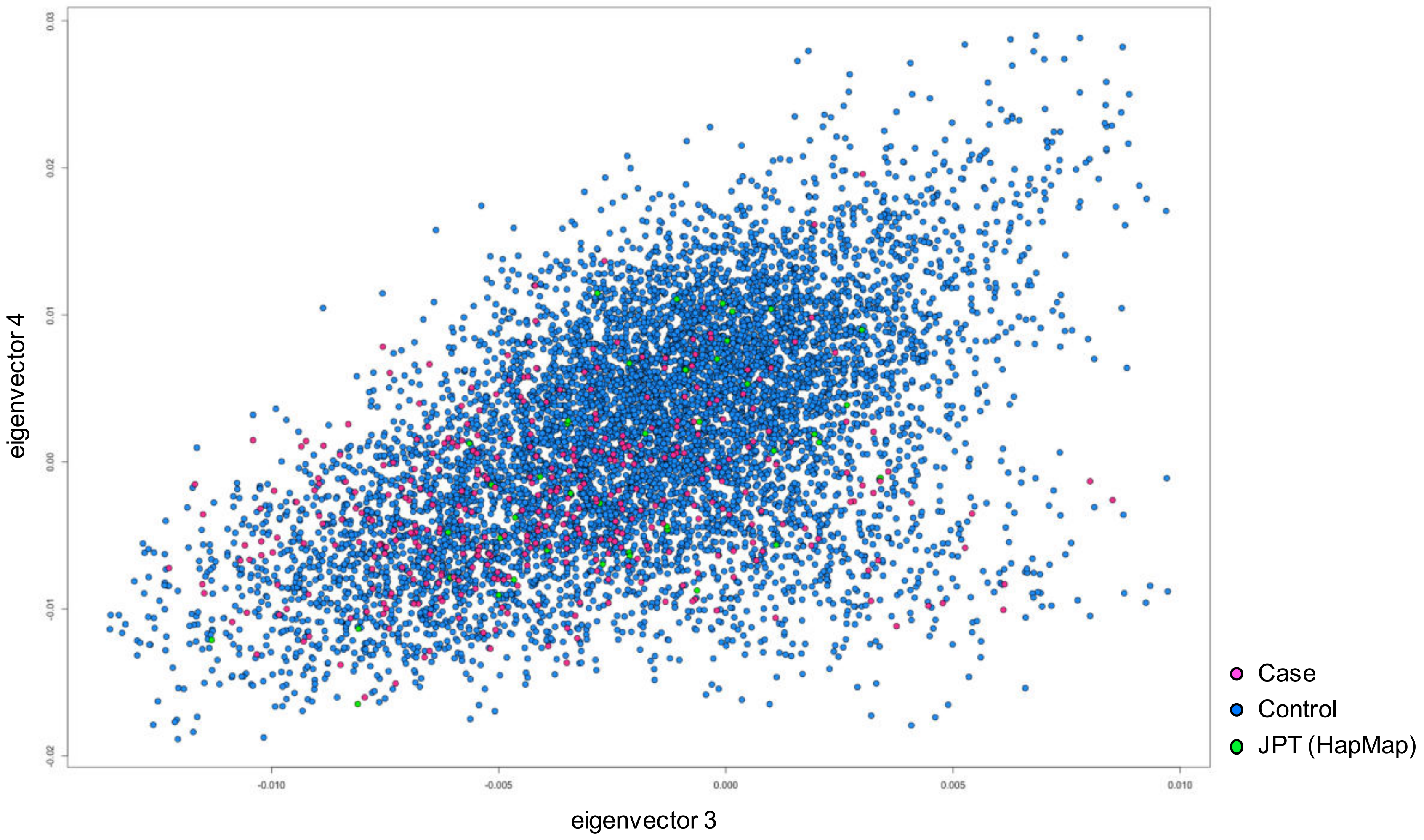

d) PCA for case, control and the four populations (JPT, CHB, CEU, and YRI of the HapMap) in the 2nd set

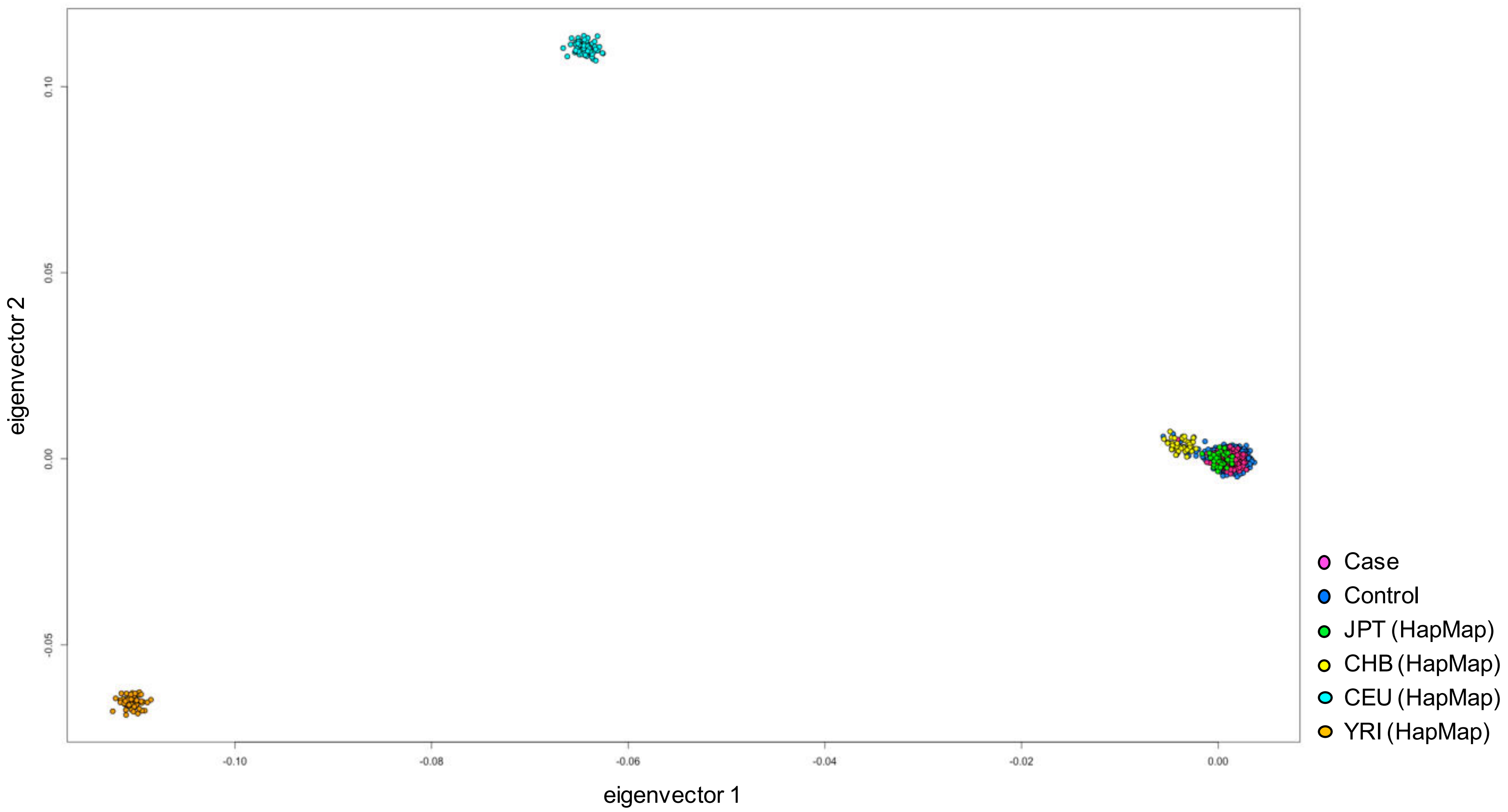

e) PCA for the East Asian population (case, control, and JPT and CHB of the HapMap) in the 2nd set

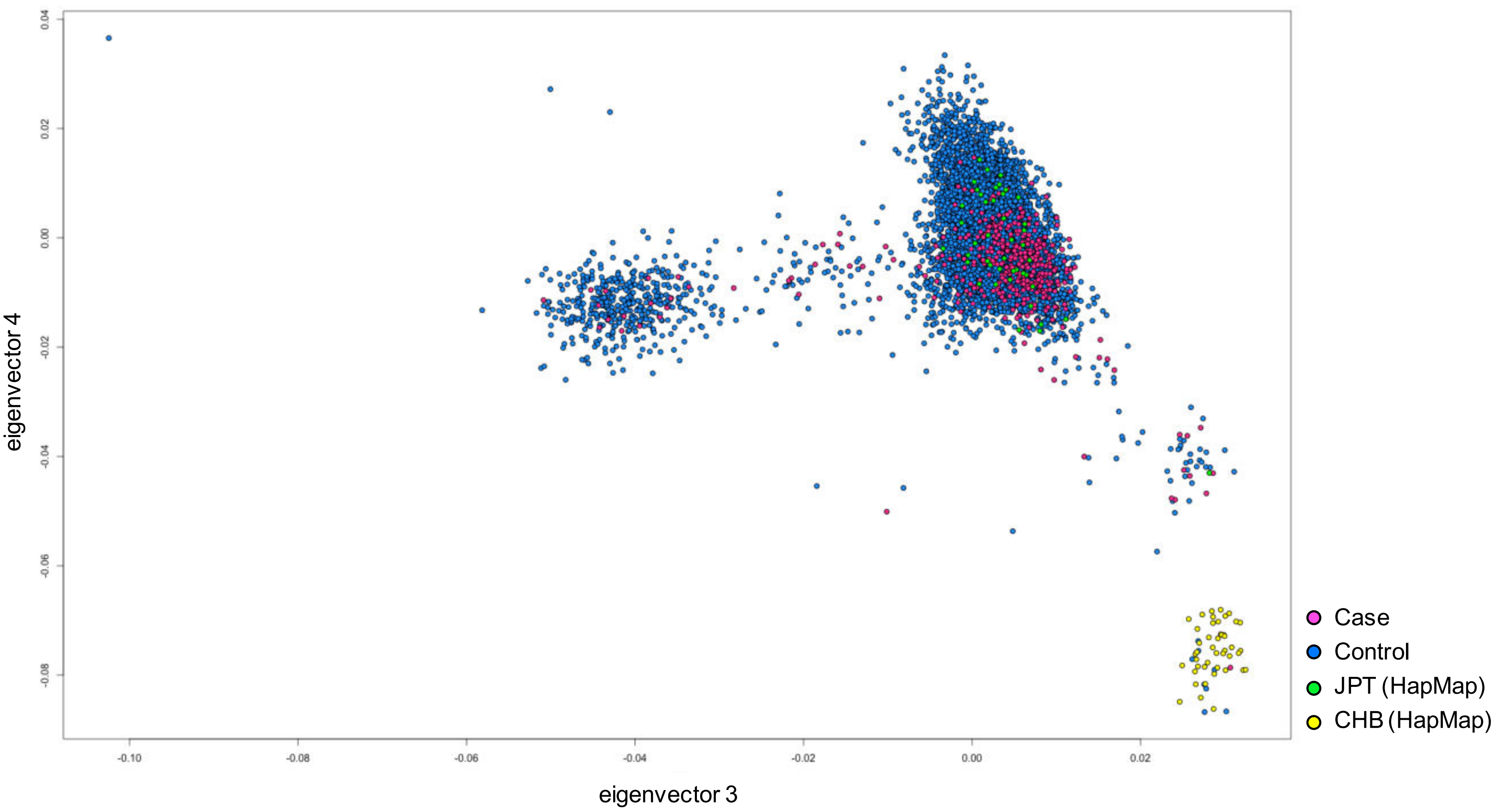

f) PCA for the Japanese cluster of the main islands in the 2nd set

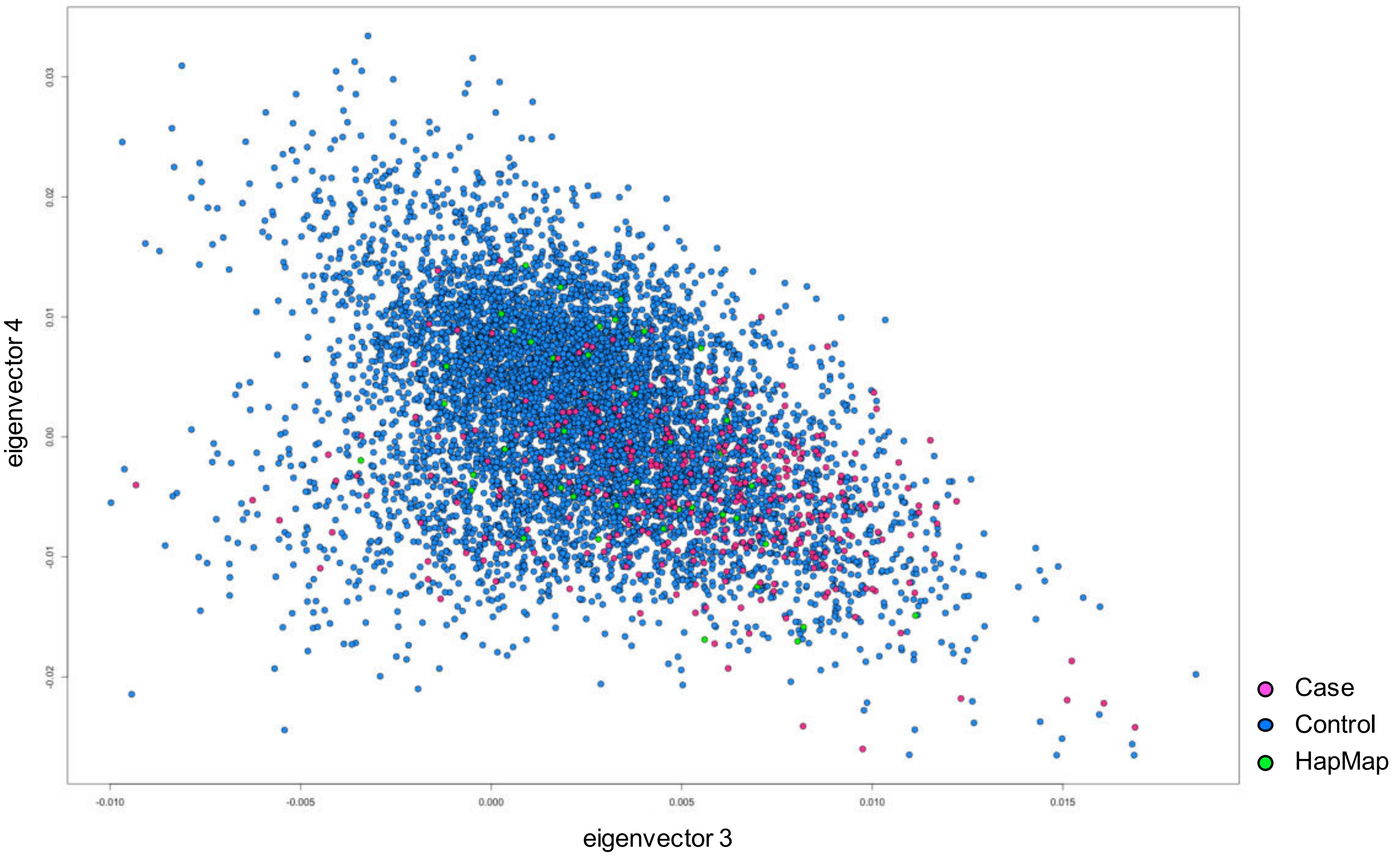

Figure S1. Results of principal component analysis (PCA)

a) PCA for case, control and the four populations (JPT, CHB, CEU, and YRI of the HapMap) in the 1st set
b) PCA for the East Asian population (case, control, and JPT and CHB of the HapMap) in the 1st set
c) PCA for the Japanese cluster of the main islands in the 1st set
d) PCA for case, control and the four populations (JPT, CHB, CEU, and YRI of the HapMap) in the 2nd set
e) PCA for the East Asian population (case, control, and JPT and CHB of the HapMap) in the 2nd set
f) PCA for the Japanese cluster of the main islands in the 2nd set

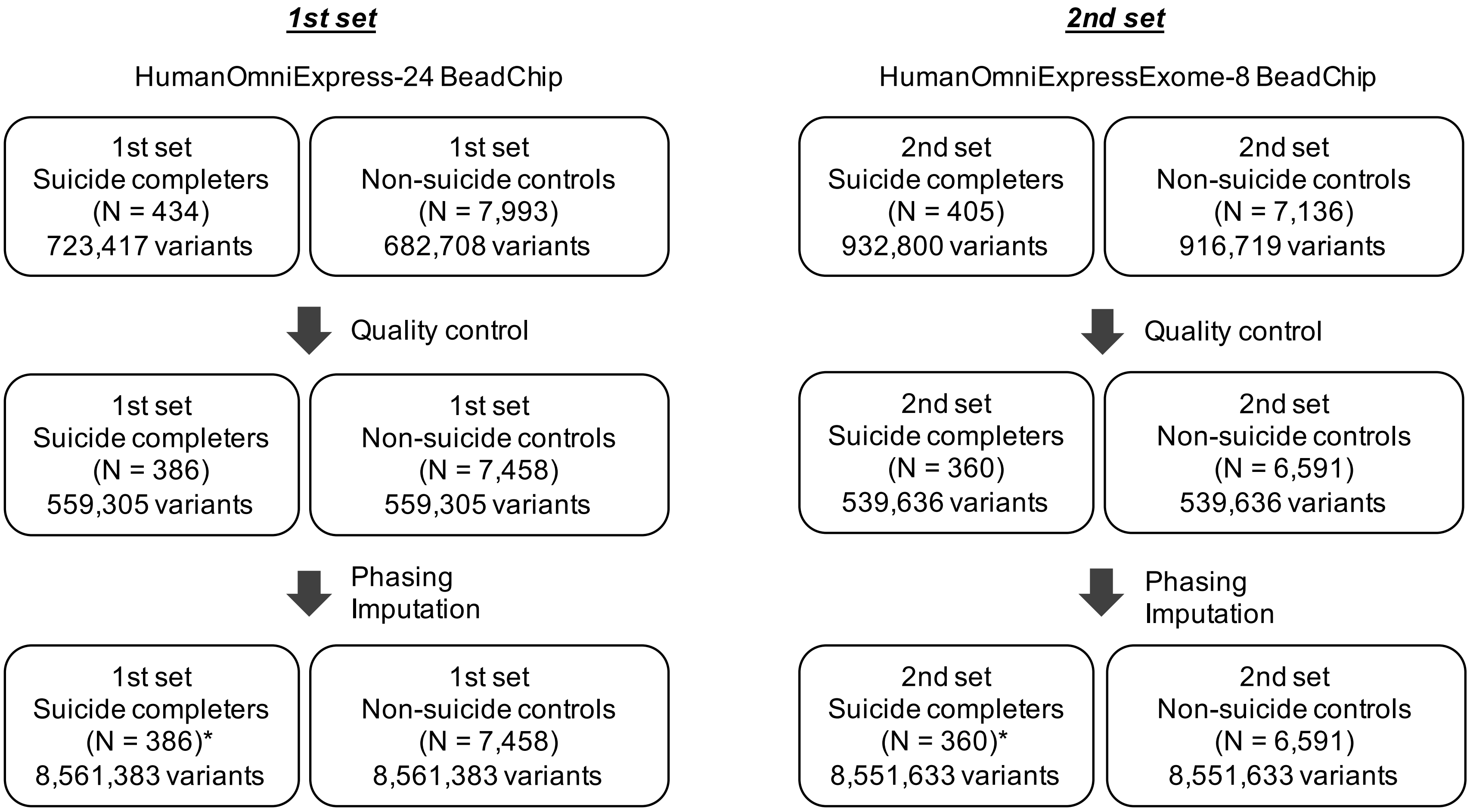

Figure S2. Overview of the study

*Accurate information for age at suicide are available only for 366 suicides and 353 suicides for the 1st set and 2nd set, respectively.

a) QQ plot of meta-analysis of 1st and 2nd set for case-control GWAS

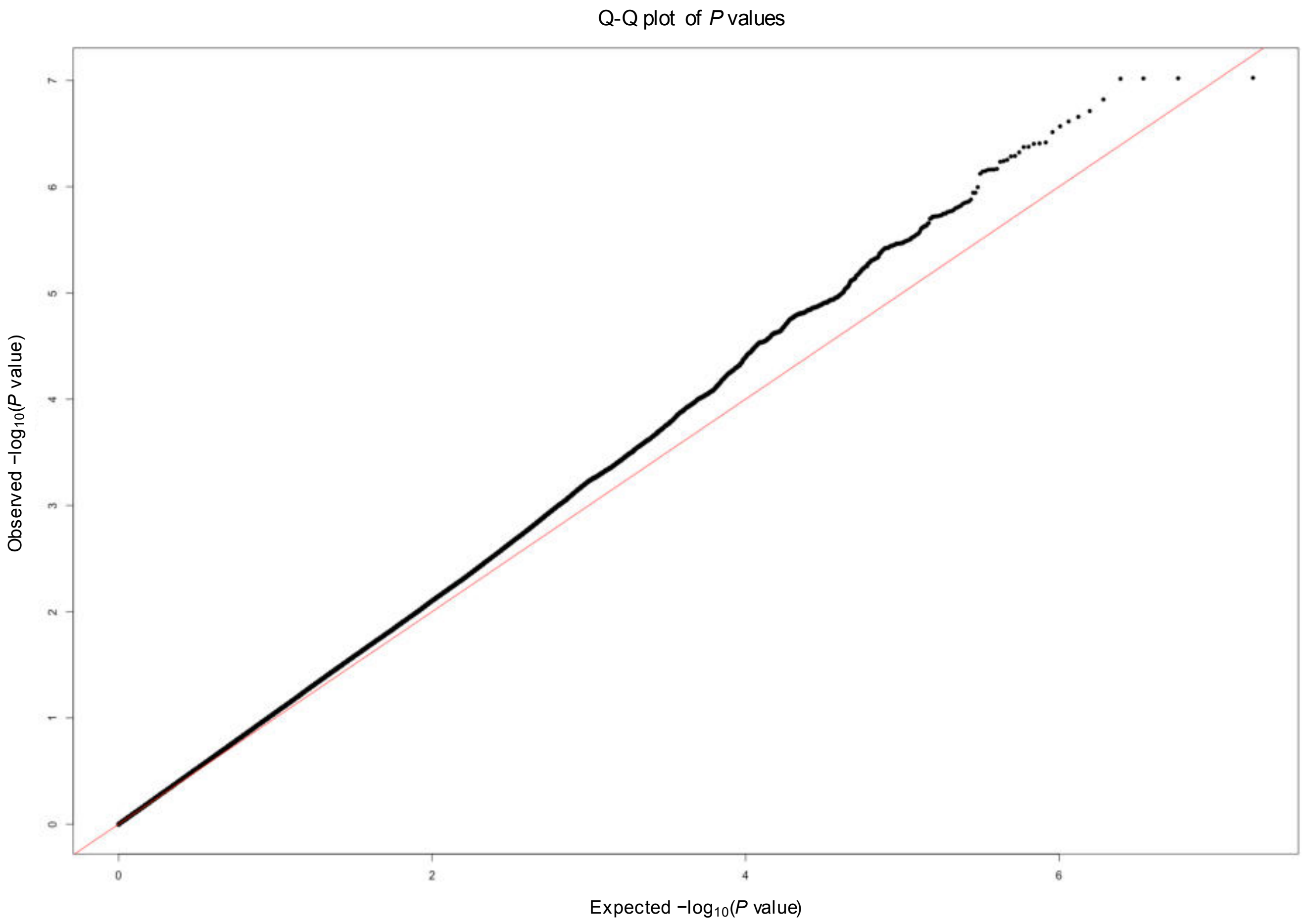

b) QQ plot of meta-analysis of 1st and 2nd set for GWAS for age at suicide

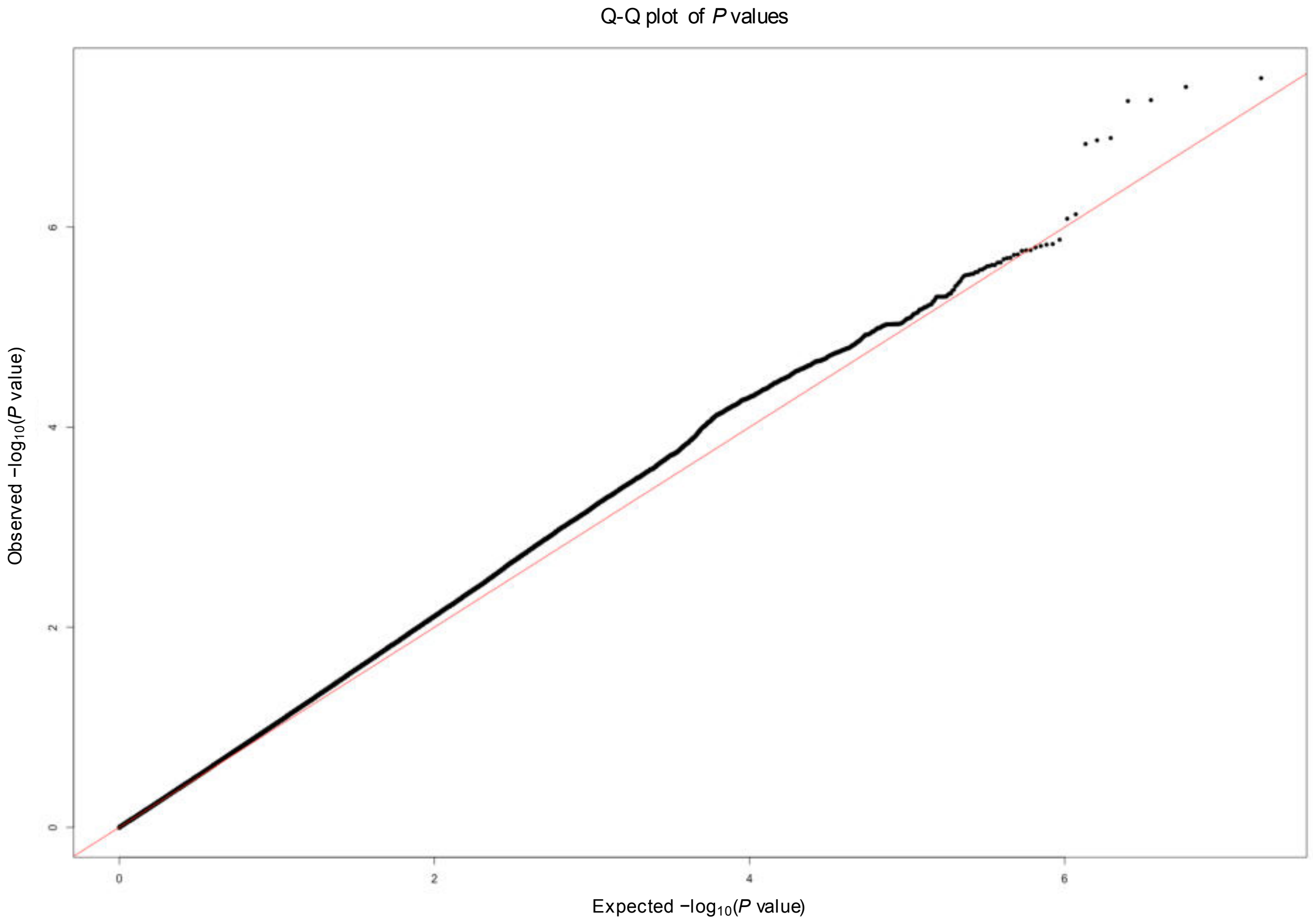

Figure S3. QQ plot of GWAS

a) QQ plot of meta-analysis of the 1st and 2nd set for case-control GWAS (lambda GC = 1.07)

b) QQ plot of meta-analysis of the 1st and 2nd set for GWAS for age at suicide (lambda GC = 1.02)

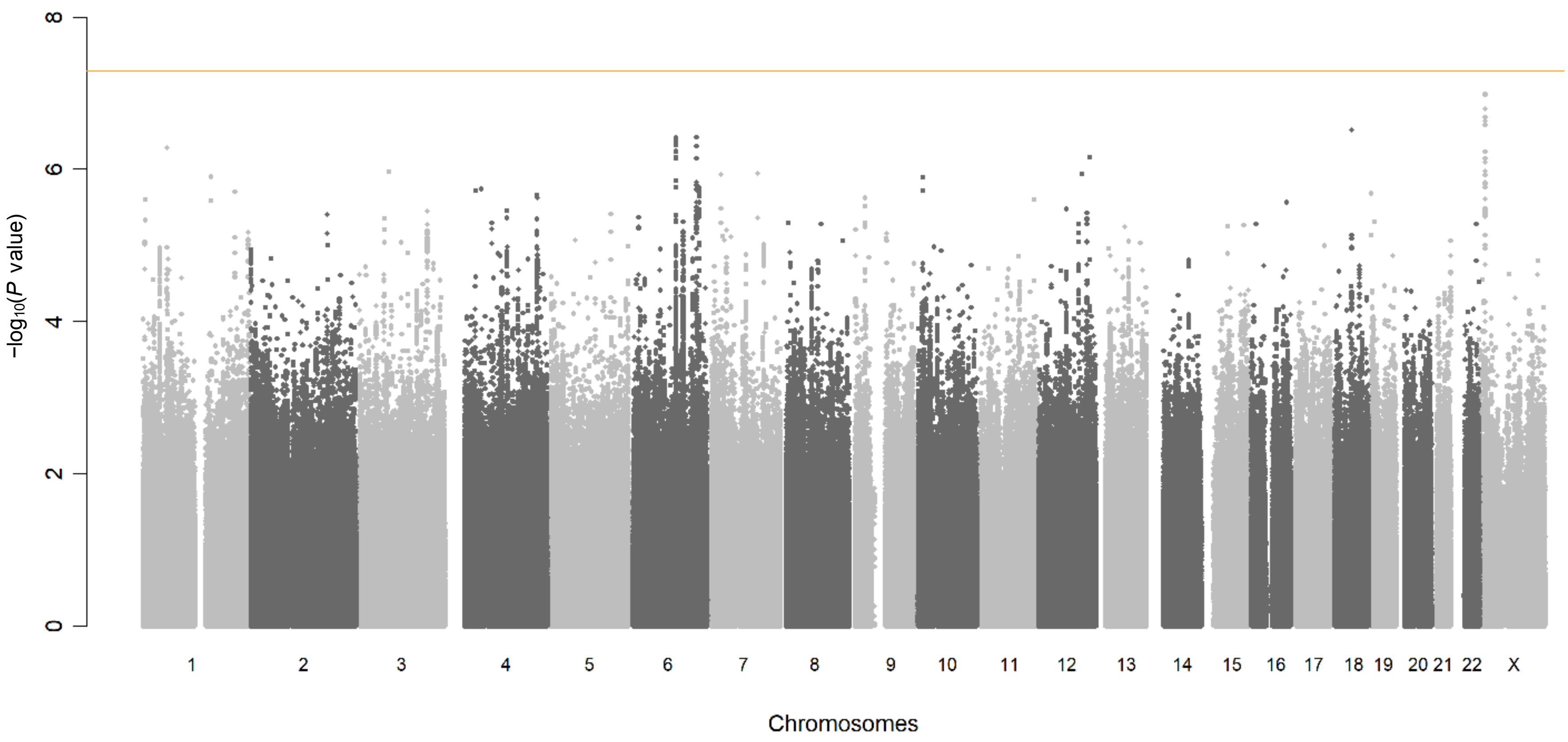

Figure S4. Manhattan plot of case-control GWAS

Manhattan plot of the meta-analysis of the 1st and 2nd set for case-control GWAS. Horizontal line indicates threshold for $P < 5.0 \times 10^{-8}$.

a)

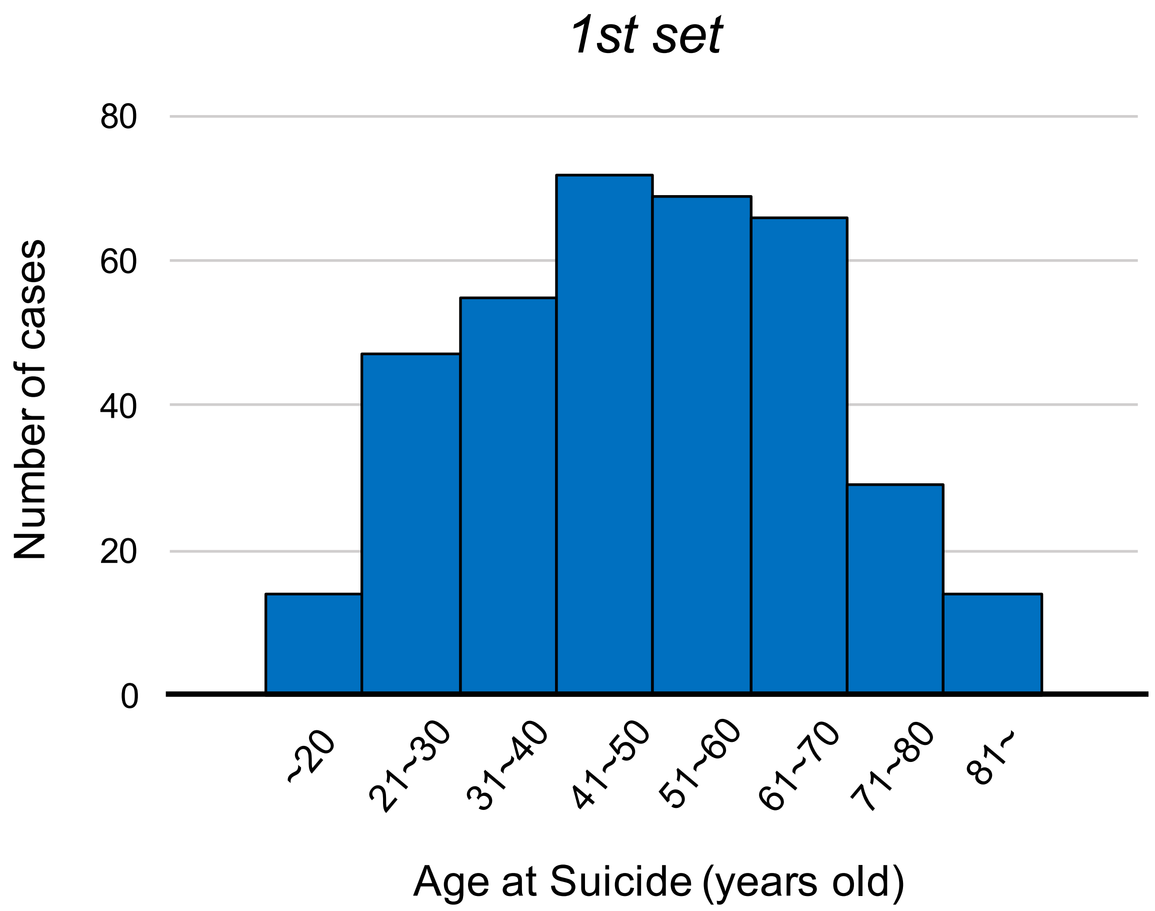

b)

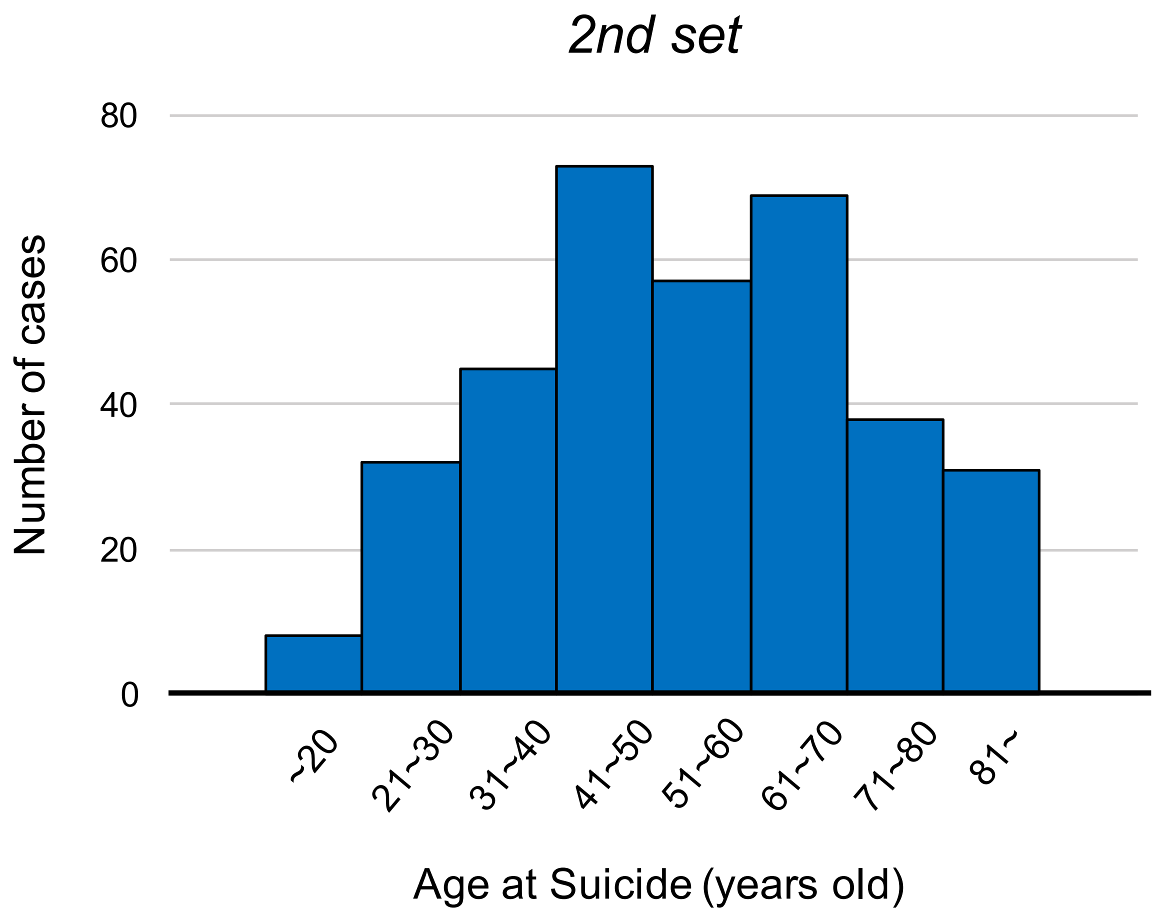

Figure S5. Histogram of age at suicide

a) Histogram of age at suicide in the 1st set

b) Histogram of age at suicide in the 2nd set

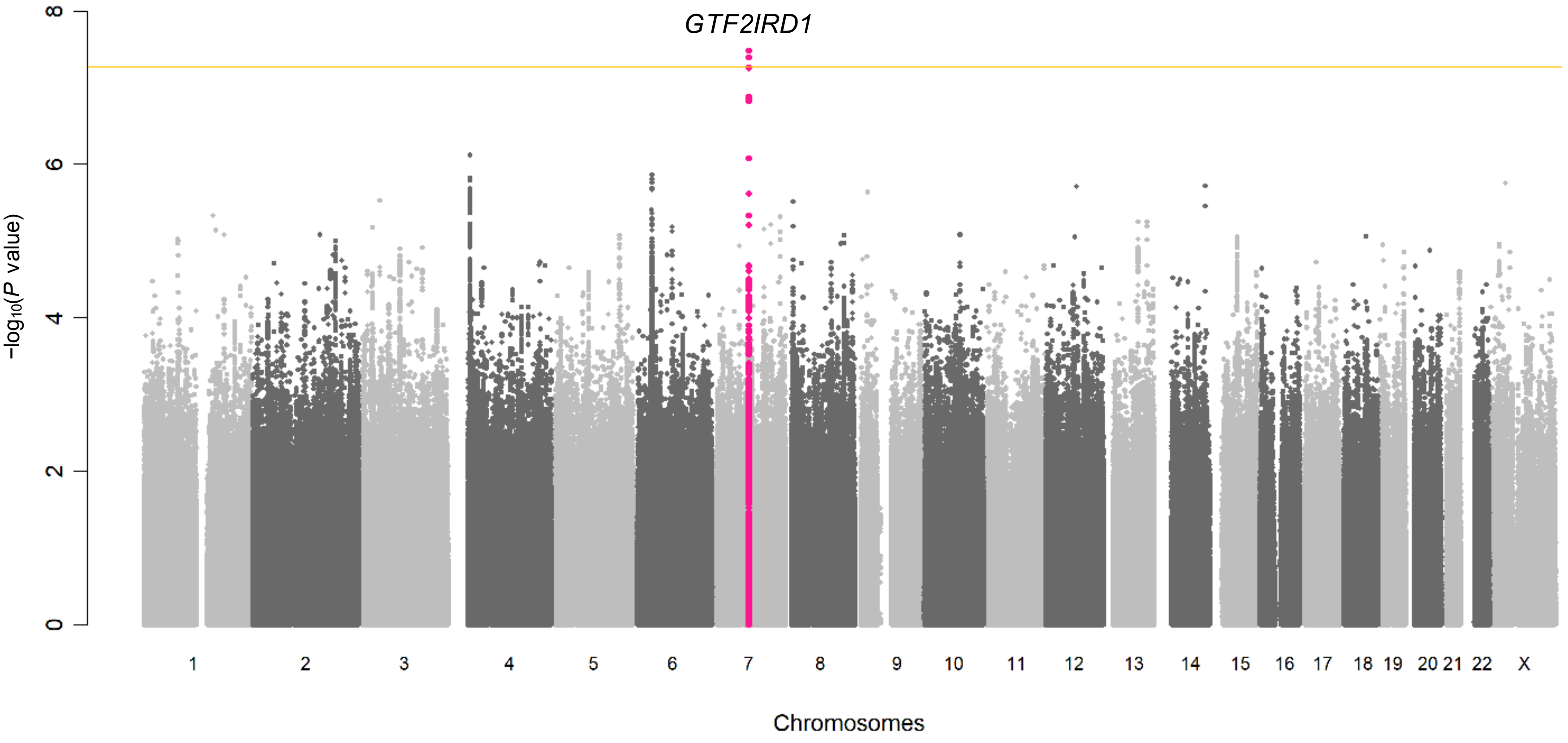

Figure S6. Manhattan plot of GWAS for age at suicide

Manhattan plot of the meta-analysis of the 1st and 2nd set for GWAS focusing age at suicide. Horizontal line indicates threshold for $P < 5.0 \times 10^{-8}$.

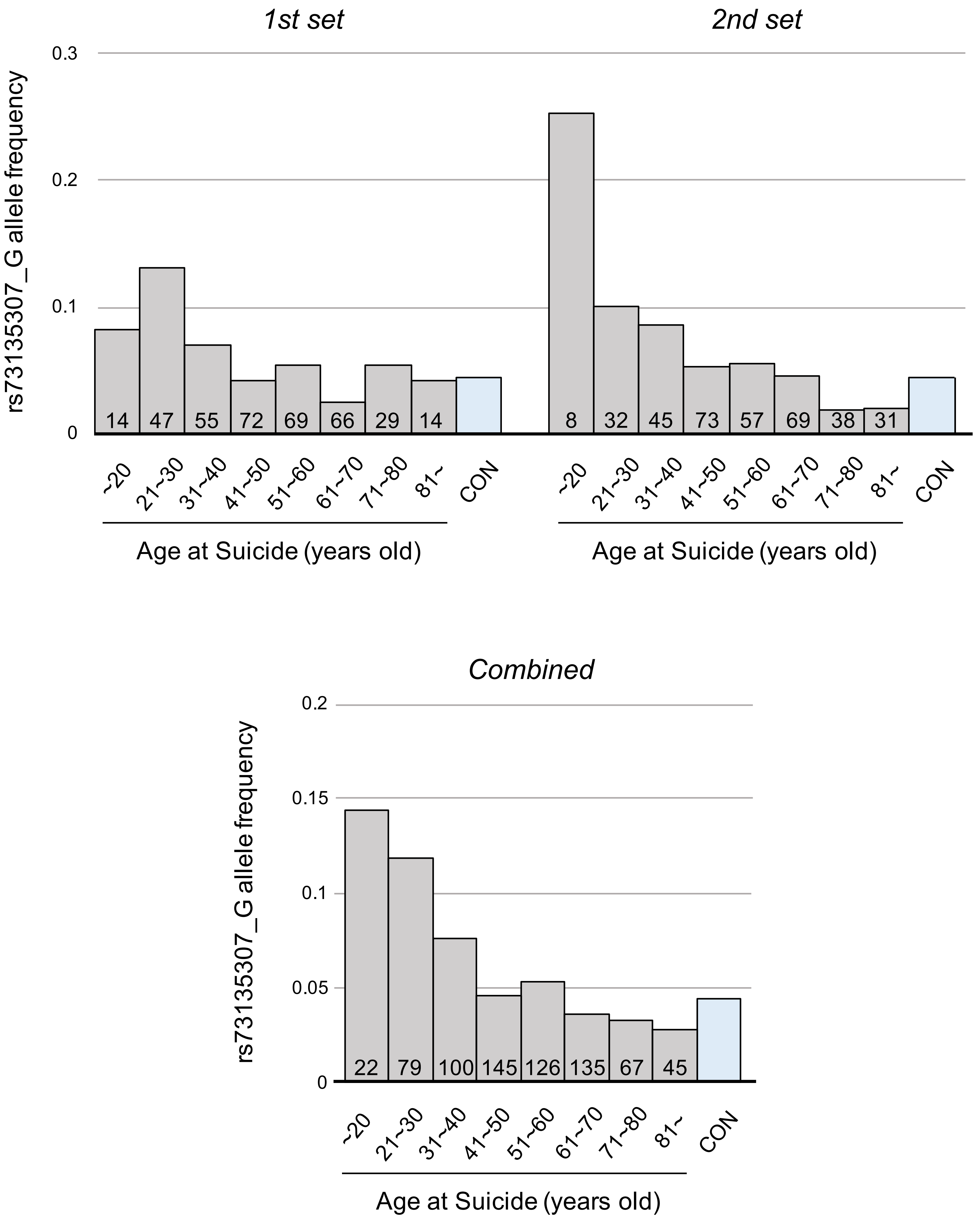

Figure S7. G allele frequency variation of rs73135307 in divided groups based on age at suicide in the 1st, 2nd, and combined set. Number of suicides are given in each bar. As references, rs73135307_G frequency of non-suicide controls (CON) in this study are also shown.
