# Supplementary material for: Genome-wide association studies identify polygenic effects for completed suicide in the Japanese population
Source: Neuropsychopharmacology. 2019 Sep 2;44(12):2119–24. doi: 10.1038/s41386-019-0506-5 (PMC6887868; doi:10.1038/s41386-019-0506-5)
Supplement: Supplementary file 2 — Supplementary Tables [file 41386_2019_506_MOESM2_ESM.docx]

**Table S1. Characteristics of subjects in the 1st set and 2nd set**

|  | 1st set | |  | 2nd set | |
| --- | --- | --- | --- | --- | --- |
|  | Suicides^a^ | Non-suicide controls^b^ |  | Suicides^a^ | Non-suicide controls^b^ |
| Number of Subjects | 386 | 7,458 |  | 360 | 6,591 |
| Male/Female | 255/131 | 4,338/3,120 |  | 239/121 | 3,481/3,110 |
| Age ± s.d. | 50.1 ± 17.6^c^ | 58.7 ± 13.4 |  | 54.0 ± 18.1^c^ | 45.2 ± 18.3 |
| Comorbid psychiatric disorders |  |  |  |  |  |
| - Mood disorders | 153 |  |  | 134 |  |
| - Psychotic disorders | 25 |  |  | 22 |  |
| - Anxiety disorders | 21 |  |  | 19 |  |
| - Personality disorders | 5 |  |  | 3 |  |
| - Alcohol/Substance use disorders | 12 |  |  | 10 |  |
| Suicide attempt history  (yes, Y; no, N; unknown, U) | Y = 83; N = 271; U = 32 |  |  | Y = 69; N = 273; U = 18 |  |
| Genotype Method | HumanOmniExpress-24 BeadChip | HumanOmniExpress-24 BeadChip |  | HumanOmniExpressExome-8 BeadChip | HumanOmniExpressExome-8 BeadChip |

^a^Individuals who died by suicide between June 1996 and July 2012 were for the 1st set, and those who died by suicide between August 2012 and February 2017 were for the 2nd set.

^b^Sample sizes of the non-suicide controls for the 1st set were; cerebral aneurysm = 1,271, esophageal cancer = 1,088, endometrial cancer = 889, COPD = 1,146, Glaucoma = 1,187, and Healthy volunteers = 1,887. Those for the 2nd set were; Epilepsy = 1,620, Urolithiasis = 980, Nephrotic syndrome = 793, Atopic dermatitis = 1,408, Graves' disease = 1,790. Subjects with non-psychiatric disorders were from the Biobank Japan project. 982 healthy volunteers were from the Osaka-Midosuji Rotary Club and 895 healthy volunteers were from the Pharma SNP consortium.

^c^Accurate information for age at suicide are available only for 366 suicides and 353 suicides for the 1st set and the 2nd set, respectively.

**Table S2. Results of the meta-analysis of our case-control GWASs (1st set and 2nd set): P_meta_ < 1.0x10^-6^**

| SNP | Chr | bp^a^ | Gene^a^  (locus) | Effect allele  (REF allele) | 1st set | | 2nd set | | Meta-analysis | | |
| --- | --- | --- | --- | --- | --- | --- | --- | --- | --- | --- | --- |
|  |  |  |  |  | *P* value | OR (95% CI) | *P* value | OR (95% CI) | *P* value | OR (95% CI) | *P*_het_^b^ |
| rs145791079 | 1 | 56443229 | – | C (T) | 8.89E−05 | 2.32  (1.52 - 3.53) | 1.58E−03 | 2.08  (1.32 – 3.28) | 5.11E−07 | 2.21  (1.62 – 3.01) | 0.73 |
| rs17058384 | 6 | 98766589 | – | T (A) | 5.06E−04 | 0.77  (0.66 – 0.89) | 2.29E−04 | 0.74  (0.63 – 0.87) | 4.19E−07 | 0.76  (0.68 – 0.84) | 0.80 |
| rs6941784 | 6 | 98767943 | – | T (C) | 4.38E−04 | 0.76  (0.66 - 0.89) | 2.46E−04 | 0.75  (0.64 – 0.87) | 3.87E−07 | 0.75  (0.68 – 0.84) | 0.83 |
| rs1395973 | 6 | 98771028 | – | C (T) | 5.52E−04 | 0.77  (0.66 - 0.89) | 2.46E−04 | 0.75  (0.64 – 0.87) | 3.79E−07 | 0.75  (0.68 – 0.84) | 0.83 |
| rs56338858 | 6 | 98773336 | – | T (A) | 4.27E−04 | 0.76  (0.65 – 0.89) | 2.74E−04 | 0.75  (0.64 – 0.87) | 4.17E−07 | 0.75  (0.68 – 0.84) | 0.85 |
| rs8372805 | 6 | 98776853 | – | T (C) | 4.26E−04 | 0.76  (0.65 – 0.89) | 3.13E−04 | 0.75  (0.64 – 0.87) | 4.72E−07 | 0.75  (0.67 – 0.84) | 0.86 |
| rs1451350 | 6 | 98780251 | – | T (G) | 4.73E−04 | 0.76  (0.65 – 0.89) | 3.48E−04 | 0.74  (0.63 – 0.88) | 5.79E−07 | 0.75  (0.67 – 0.84) | 0.87 |
| rs62420599 | 6 | 98780531 | – | C (T) | 4.26E−04 | 0.76  (0.65 – 0.88) | 3.84E−04 | 0.75  (0.63 – 0.88) | 5.71E−07 | 0.75  (0.67 – 0.84) | 0.90 |
| rs1078326 | 6 | 98785243 | – | A (G) | 4.24E−04 | 0.75  (0.64 – 0.88) | 4.85E−04 | 0.75  (0.63 – 0.88) | 7.10E−07 | 0.75  (0.67 – 0.84) | 0.93 |
| rs59291342 | 6 | 146475333 | *GRM1*  (intronic) | G (A) | 8.57E−05 | 1.62  (1.28 – 2.08) | 2.23E−03 | 1.51  (1.16 – 1.97) | 7.03E−07 | 1.57  (1.32 – 1.88) | 0.68 |
| rs6913400 | 6 | 146479408 | *GRM1*  (intronic) | C (G) | 3.16E−05 | 1.68  (1.32 – 2.15) | 2.72E−03 | 1.51  (1.15 – 1.97) | 3.75E−07 | 1.60  (1.33 – 1.91) | 0.56 |
| rs56116136 | 6 | 146492316 | *GRM1*  (intronic) | T (C) | 4.53E−05 | 1.67  (1.30 – 2.13) | 2.77E−03 | 1.51  (1.15 – 1.97) | 4.92E−07 | 1.59  (1.33 – 1.90) | 0.59 |
| rs11069071 | 12 | 119200350 | – | T (C) | 1.65E−04 | 1.32  (1.14 – 1.53) | 1.18E−03 | 1.29  (1.11 – 1.51) | 6.84E−07 | 1.31  (1.18 – 1.46) | 0.81 |
| rs11069079 | 12 | 119213948 | – | G (A) | 2.89E−04 | 1.32  (1.14 – 1.54) | 6.94E−04 | 1.32  (1.12 – 1.56) | 6.90E−07 | 1.32  (1.18 – 1.48) | 1.00 |
| rs500042 | 18 | 38507299 | – | A (T) | 2.68E−03 | 1.37  (1.12 – 1.68) | 1.60E−05 | 1.64  (1.31 – 2.06) | 3.02E−07 | 1.49  (1.28 – 1.73) | 0.24 |
| rs5980312 | X | 16694826 | *CTPS2*  (intronic) | C (A) | 5.38E−04 | 1.58  (1.22 – 2.05) | 4.46E−05 | 1.76  (1.34 – 2.31) | 1.03E−07 | 1.66  (1.38 – 2.01) | 0.58 |
| rs5980314 | X | 16696005 | *CTPS2*  (intronic) | G (A) | 5.38E−04 | 1.58  (1.22 – 2.04) | 4.43E−05 | 1.76  (1.34 – 2.30) | 1.02E−07 | 1.66  (1.38 – 2.00) | 0.58 |
| rs6527702 | X | 16696117 | *CTPS2*  (intronic) | G (T) | 5.37E−04 | 1.58  (1.22 – 2.04) | 4.42E−05 | 1.76  (1.34 – 2.30) | 1.01E−07 | 1.66  (1.38 – 2.00) | 0.58 |
| rs7049850 | X | 16697183 | *CTPS2*  (intronic) | T (C) | 5.34E−04 | 1.58  (1.22 – 2.05) | 4.40E−05 | 1.76  (1.34 – 2.30) | 9.96E−08 | 1.66  (1.38 – 2.00) | 0.58 |
| rs4831050 | X | 16700128 | *CTPS2*  (intronic) | T (A) | 6.40E−04 | 1.57  (1.21 – 2.03) | 5.97E−05 | 1.74  (1.33 – 2.28) | 1.59E−07 | 1.65  (1.37 – 1.99) | 0.59 |
| rs5980319 | X | 16701765 | *CTPS2*  (intronic) | G (A) | 7.15E−04 | 0.64  (0.49 – 0.83) | 6.95E−05 | 0.58  (0.44 – 0.76) | 2.04E−07 | 0.61  (0.51 – 0.74) | 0.60 |
| rs5978787 | X | 16702128 | *CTPS2*  (intronic) | G (A) | 7.30E−04 | 1.57  (1.21 – 2.03) | 7.80E−05 | 1.73  (1.32 – 2.26) | 2.30E−07 | 1.64  (1.36 – 1.98) | 0.61 |
| rs5980321 | X | 16702697 | *CTPS2*  (intronic) | T (A) | 7.49E−04 | 0.64  (0.49 – 0.83) | 8.44E−05 | 0.58  (0.44 – 0.76) | 2.56E−07 | 0.61  (0.51 – 0.74) | 0.62 |
| rs4830556 | X | 16703797 | *CTPS2*  (intronic) | C (T) | 1.53E−03 | 0.66  (0.51 – 0.85) | 1.30E−04 | 0.59  (0.45 – 0.77) | 7.93E−07 | 0.63  (0.52 – 0.75) | 0.57 |
| rs6527703 | X | 16706501 | *CTPS2*  (intronic) | C (T) | 1.12E−03 | 1.55  (1.19 – 2.01) | 1.34E−04 | 1.70  (1.29 – 2.23) | 5.80E−07 | 1.62  (1.34 – 1.95) | 0.63 |

Loci that reached *P* < 1.0 × 10^−6^ after meta-analysis of the 1st and 2nd sets are shown. Chr, chromosome; bp, base pair; REF, reference allele.

^a^Positions and genes are based on Human Genome version 19 (hg19; build 37).

^b^*P* for heterogeneity between two analyses (1st set and 2nd set) were calculated by Cochran’s Q test.

**Table S3. Case-control GWAS results for previously implicated SNPs in prior GWAS of suicidal behavior**

| Reference^a^  Phenotype  *P* value | Index SNP^a^ | Chr | bp^b^ | Effect allele  (REF allele) | 1st set | | 2nd set | | Meta-analysis | | |
| --- | --- | --- | --- | --- | --- | --- | --- | --- | --- | --- | --- |
|  |  |  |  |  | *P* value | OR (95% CI) | *P* value | OR (95% CI) | *P* value | OR (95% CI) | *P*_het_^c^ |
| Willour et al.^1^  SI  5.07E−08 | rs300774 | 2 | 112496 | C (A) | 0.370 | 1.11  (0.88 – 1.39) | 0.013 | 1.39  (1.07 – 1.79) | 0.020 | 1.22  (1.03 – 1.45) | 0.20 |
| Levey et al.^2^  SA  4.48E-07 | rs16860543 | 2 | 173351176 | A (G) | 0.702 | 1.03  (0.89 – 1.19) | 0.896 | 0.99  (0.85 – 1.16) | 0.851 | 1.01  (0.91 – 1.12) | 0.72 |
| Kimbrel et al.^3^  SI  1.88E−07 | rs2613142 | 4 | 43848424 | G (A) | 0.100 | 0.86  (0.71 – 1.03) | 0.273 | 1.11  (0.65 – 1.90) | 0.603 | 0.96  (0.84 – 1.11) | 0.06 |
| Erlangsen et al.^4^  SA  5.44E−08 | rs6880062 | 5 | 153298025 | G (A) | 0.947 | 1.01  (0.86 – 1.17) | 0.023 | 0.83  (0.70 – 0.98) | 0.131 | 0.92  (0.82 – 1.03) | 0.09 |
| Stein et al.^5^  SA  5.24E−10 | rs12524136 | 6 | 84935441 | T (C) | – | – | 0.496 | 0.70  (0.26 – 1.93) | – | – | – |
| Kimbrel et al.^3^  SA  7.58E−07 | rs11762112 | 7 | 27526292 | G (A) | 0.138 | 1.13  (0.96 – 1.34) | 0.140 | 1.14  (0.96 – 1.36) | 0.036 | 1.13  (1.00 – 1.28) | 0.95 |
| Galfalvy et al.^6^  SC  2.00E−07 | rs336284 | 7 | 35293972 | G (A) | 0.461 | 0.95  (0.82 – 1.10) | 0.514 | 0.95  (0.80 – 1.12) | 0.325 | 0.95  (0.85 – 1.06) | 1.00 |
| Mullins et al.^7^  SI  2.41E−07 | rs17173608 | 7 | 150036664 | G (T) | 0.906 | 0.98  (0.74 – 1.31) | 0.364 | 1.15  (0.85 – 1.55) | 0.584 | 1.06  (0.86 – 1.30) | 0.46 |
| Erlangsen et al.^4^  SA  8.40E−07 | rs76426299 | 8 | 58350711 | A (G) | 0.802 | 1.03  (0.82 – 1.29) | 0.235 | 1.16  (0.91 – 1.47) | 0.318 | 1.09  (0.92 – 1.28) | 0.49 |
| Erlangsen et al.^4^  SA  9.80E−07 | rs7862648 | 9 | 18290857 | G (A) | 0.432 | 1.21  (0.76 – 1.92) | 0.638 | 0.87  (0.48 – 1.57) | 0.743 | 1.06  (0.74 – 1.54) | 0.39 |
| Perroud et al.^8^  SI  8.28E−07 | rs11143230 | 9 | 74887703 | C (A) | 0.073 | 0.83  (0.67 – 1.02) | 0.953 | 1.01  (0.82 – 1.23) | 0.222 | 0.91  (0.79 – 1.06) | 0.19 |
| Levey et al.^2^  SA  4.73E-07 | rs12683724 | 9 | 83116342 | A (G) | 0.622 | 1.04  (0.89 – 1.21) | 0.434 | 1.06  (0.91 – 1.25) | 0.371 | 1.05  (0.94 – 1.17) | 0.82 |
| Levey et al.^2^  SA  8.53E-07 | rs76477759 | 9 | 105349079 | C (G) | 0.675 | 0.95  (0.75 – 1.21) | 0.544 | 1.08  (0.85 – 1.36) | 0.891 | 0.99  (0.84 – 1.17) | 0.47 |
| Levey et al.^2^  SA  2.38E-07 | rs76182109 | 11 | 96032273 | A (G) | 0.454 | 1.07  (0.90 – 1.27) | 0.857 | 1.02  (0.85 – 1.22) | 0.504 | 0.96  (0.84 – 1.09) | 0.70 |
| Strawbridge et al.^9^  SI  1.07E-08 | rs598046 | 11 | 99516468 | T (G) | 0.685 | 0.97  (0.84 – 1.12) | 0.377 | 0.93  (0.79 – 1.09) | 0.369 | 0.95  (0.85 – 1.06) | 0.71 |
| Levey et al.^2^  SA  1.07E-08 | rs1677091 | 12 | 21780851 | A (C) | 0.848 | 1.02  (0.81 – 1.29) | 0.641 | 1.06  (0.83 – 1.35) | 0.645 | 0.96  (0.81 – 1.14) | 0.84 |
| Levey et al.^2^  SA  2.07E-08 | rs683813 | 12 | 27600331 | T (C) | 0.249 | 1.11  (0.93 – 1.33) | 0.576 | 0.95  (0.79 – 1.14) | 0.665 | 1.03  (0.91 – 1.17) | 0.23 |
| Erlangsen et al.^4^  SA  4.55E−07 | rs112595860 | 12 | 32640591 | G (C) | 0.520 | 0.90  (0.66 – 1.23) | 0.562 | 0.91  (0.65 – 1.26) | 0.387 | 0.99  (0.79 – 1.24) | 0.99 |
| Levey et al.^2^  SA  3.01E-07 | rs113498843 | 13 | 50967576 | A (G) | 0.181 | 0.57  (0.25 – 1.30) | 0.071 | 0.39  (0.14 – 1.08) | 0.030 | 0.49  (0.26 – 0.93) | 0.58 |
| Strawbridge et al.^9^  SI  3.49E-08 | rs7989250 | 13 | 64900801 | A (C) | 0.007 | 0.76  (0.62 – 0.92) | 0.218 | 0.87  (0.70 – 1.09) | **0.004** | 0.81  (0.69 – 0.94) | 0.37 |
| Kimbrel et al.^3^  SI  9.88E−07 | rs12100626 | 14 | 75091805 | T (C) | 0.065 | 0.78  (0.60 – 1.02) | 0.862 | 0.98  (0.75 – 1.28) | 0.149 | 0.87  (0.72 – 1.05) | 0.24 |
| Levey et al.^2^  SA  2.36E-08 | rs72740082 | 15 | 80508139 | A (T) | 0.435 | 1.08  (0.89 – 1.33) | 0.551 | 1.07  (0.94 – 1.22) | 0.328 | 0.93  (0.80 – 1.08) | 0.92 |
| Levey et al.^2^  SA  7.91E-07 | rs9927702 | 16 | 24558653 | A (G) | 0.133 | 0.87  (0.73 – 1.04) | 0.779 | 1.03  (0.85 – 1.23) | 0.376 | 1.06  (0.93 – 1.20) | 0.21 |
| Galfalvy et al.^6^  SI  8.31E−07 | rs6055685 | 20 | 8213786 | A (G) | 0.059 | 1.19  (0.99 – 1.43) | 0.656 | 0.95  (0.78 – 1.17) | 0.266 | 1.08  (0.94 – 1.24) | 0.11 |
| Erlangsen et al.^4^  SA  2.80E−08 | rs4809706 | 20 | 47193719 | G (A) | 0.693 | 0.97  (0.83 – 1.13) | 0.427 | 0.94  (0.80 – 1.10) | 0.406 | 0.95  (0.86 – 1.07) | 0.76 |
| Erlangsen et al.^4^  SA  6.78E−07 | rs117090422^d^  (rs150801052) | 22 | 36242779 | A (G) | 0.473 | 0.77  (0.37 – 1.58) | 0.524 | 0.80  (0.41 – 1.58) | 0.339 | 0.79  (0.48 – 1.29) | 0.93 |

Loci that reached *P* < 1.0 × 10^−6^ reported in previous GWASs and MAF > 0.01 in JPT population (the 1000 Genomes Project phase 3) were selected. A locus that reached statistical significance for our replication analysis (*P* < 0.05) with the same direction of the allelic effect is shown in bold. Chr, chromosome; bp, base pair; REF, reference allele; SI, suicide ideation; SA, suicide attempt; SC; suicide completion.

^a^Only the top SNP in the same region in each reference was selected.

^b^Positions are based on Human Genome version 19 (hg19), build 37.

^c^*P* for heterogeneity between two analyses (1st set and 2nd set) were calculated by Cochran’s Q test.

^d^rs117090422 is a proxy SNP with *r*^2^ > 0.8 for rs150801052 in East Asian samples of the 1000 Genomes Project phase 3.

**References**

1. Willour VL, et al. A genome-wide association study of attempted suicide. *Mol Psychiatry.* 2012; **17**: 433–44.
2. Levey DF, et al. Genetic associations with suicide attempt severity and genetic overlap with major depression. *Transl Psychiatry.* 2019; **9**: 22.
3. Kimbrel NA, et al. A genome-wide association study of suicide attempts and suicidal ideation in U.S. military veterans. *Psychiatry Res.* 2018; **269**: 64–9.
4. Erlangsen A, et al. Genetics of suicide attempts in individuals with and without mental disorders: a population-based genome-wide association study. *Mol Psychiatry.* 2018 [Epub ahead of print].
5. Stein MB, et al. [Genomewide association studies of suicide attempts in US soldiers.](https://www.ncbi.nlm.nih.gov/pubmed/28902444) *Am J Med Genet B Neuropsychiatr Genet.* 2017; **174**: 786–97.
6. Galfalvy H, et al. A genome-wide association study of suicidal behavior. *Am J Med Genet B Neuropsychiatr Genet.* 2015; **168**: 557–63.
7. Mullins N, et al. Genetic relationships between suicide attempts, suicidal ideation and major psychiatric disorders: a genome-wide association and polygenic scoring study. *Am J Med Genet B Neuropsychiatr Genet.* 2014; **165B**: 428–37.
8. Perroud N, et al. Genome-wide association study of increasing suicidal ideation during antidepressant treatment in the GENDEP project. *Pharmacogenomics J.* 2012; **12**: 68–77.
9. Strawbridge RJ, et al. Identification of novel genome-wide associations for suicidality in UK Biobank, genetic correlation with psychiatric disorders and polygenic association with completed suicide. *EBioMedicine.* 2019; pii: S2352-3964: 30077–5.

**Table S4. Results of the polygenic risk score (PRS) analysis for our case-control GWASs (1st set and 2nd set)**

| Discovery | Target | *P*_t_ = 0.1 | | | *P*_t_ = 0.2 | | | *P*_t_ = 0.3 | | | *P*_t_ = 0.4 | | | *P*_t_ = 0.5 | | |
| --- | --- | --- | --- | --- | --- | --- | --- | --- | --- | --- | --- | --- | --- | --- | --- | --- |
|  |  | N of SNPs | R^2^ | *P* | N of SNPs | R^2^ | *P* | N of SNPs | R^2^ | *P* | N of SNPs | R^2^ | *P* | N of SNPs | R^2^ | *P* |
| 1st set | 2nd set | 18,626 | 0.013 | 1.7E-08 | 31,794 | 0.017 | 5.2E-10 | 42,954 | 0.021 | 3.0E-11 | 52,295 | 0.024 | 1.6E-12 | 60,433 | 0.023 | 3.6E-12 |
| 2nd set | 1st set | 19,148 | 0.015 | 8.4E-10 | 32,313 | 0.020 | 5.1E-12 | 43,333 | 0.022 | 1.1E-12 | 52,535 | 0.023 | 2.7E-13 | 60,496 | 0.022 | 1.0E-12 |

*P*_t_: *P* threshold, R^2^: Nagelkerke’s R^2^

**Table S5. Distribution of background information for completed suicide in three age groups for the 1st set and 2nd set**

|  | 1st set | | |  | 2nd set | | |
| --- | --- | --- | --- | --- | --- | --- | --- |
| Age at suicide (years old) | ~30 | 31~50 | 51~ |  | ~30 | 31~50 | 51~ |
| Number of Subjects | 61 | 127 | 178 |  | 40 | 118 | 195 |
| Background information  for completed suicide |  |  |  |  |  |  |  |
| - Understandable psychological factors (e.g. jobs, poverty, family dissension, exacerbation of psychiatric disorders) | 37 | 96 | 104 |  | 24 | 101 | 115 |
| - Severe physical disease | 0 | 9 | 52 |  | 0 | 5 | 59 |

**Table S6. Results of the meta-analysis of age at completed suicide (1st set and 2nd set): *P*_meta_ < 1.0 x 10^−6^**

| SNP | Chr | Position^a^  (bp) | Gene^a^  (locus) | Effect allele  (REF allele) | 1st set | | | 2nd set | | | Meta-analysis | | | |
| --- | --- | --- | --- | --- | --- | --- | --- | --- | --- | --- | --- | --- | --- | --- |
|  |  |  |  |  | beta | s.e. | *P* value | beta | s.e. | *P* value | beta | s.e. | *P* value | *P*_het_^b^ |
| rs73135307 | 7 | 73869857 | *GTF2IRD1*  (intronic) | G (C) | −9.84 | 2.97 | 9.17E−04 | −15.6 | 3.39 | 4.15E−06 | −12.3 | 2.23 | **3.25E−08** | 0.20 |
| rs67059828 | 7 | 73876693 | *GTF2IRD1*  (intronic) | C (T) | −9.63 | 2.82 | 6.38E−04 | −14.7 | 3.30 | 8.34E−06 | −11.8 | 2.14 | **3.98E−08** | 0.24 |
| rs73135312 | 7 | 73874239 | *GTF2IRD1*  (intronic) | A (G) | −9.38 | 2.90 | 1.24E−03 | −15.3 | 3.35 | 4.75E−06 | −11.9 | 2.19 | 5.39E−08 | 0.18 |
| rs73135316 | 7 | 73875232 | *GTF2IRD1*  (intronic) | G (A) | −9.35 | 2.90 | 1.25E−03 | −15.3 | 3.34 | 4.80E−06 | −11.9 | 2.19 | 5.51E−08 | 0.18 |
| rs6963457 | 7 | 73892466 | *GTF2IRD1*  (intronic) | A (G) | −8.64 | 2.77 | 1.80E−03 | −13.9 | 3.13 | 8.93E−06 | −11.0 | 2.07 | 1.29E−07 | 0.21 |
| rs145424631 | 7 | 73882930 | *GTF2IRD1*  (intronic) | C (A) | −9.31 | 3.07 | 2.39E−03 | −15.3 | 3.40 | 6.67E−06 | −12.0 | 2.28 | 1.36E−07 | 0.19 |
| rs80243771 | 7 | 73751060 | *CLIP2*  (intronic) | G (A) | −11.3 | 3.40 | 8.65E−04 | −13.7 | 3.34 | 4.23E−05 | −12.5 | 2.38 | 1.48E−07 | 0.62 |
| rs546172928 | 4 | 5318174 | *STK32B*  (intronic) | T (C) | −8.14 | 1.71 | 1.99E−06 | −3.98 | 1.85 | 3.18E−02 | −6.23 | 1.26 | 7.46E−07 | 0.10 |

Loci that reached *P* < 5.0 × 10^−8^ after meta-analysis of the 1st and 2nd set are shown in bold. Chr, chromosome; bp, base pair; REF, reference allele.

^a^Positions and genes are based on Human Genome version 19 (hg19), build 37.

^b^*P* for heterogeneity between two analyses (1st set and 2nd set) were calculated by Cochran’s Q test.

**Table S7. Results of the PRS analysis of GWAS for age at completed suicide (1st set and 2nd set)**

| Discovery | Target | *P*_t_ = 0.1 | | | *P*_t_ = 0.2 | | | *P*_t_ = 0.3 | | | *P*_t_ = 0.4 | | | *P*_t_ = 0.5 | | |
| --- | --- | --- | --- | --- | --- | --- | --- | --- | --- | --- | --- | --- | --- | --- | --- | --- |
|  |  | N of SNPs | R^2^ | *P* | N of SNPs | R^2^ | *P* | N of SNPs | R^2^ | *P* | N of SNPs | R^2^ | *P* | N of SNPs | R^2^ | *P* |
| 1st set | 2nd set | 18,179 | 8.4E-04 | 0.59 | 31,184 | 7.8E-04 | 0.60 | 42,053 | 1.6E-03 | 0.45 | 51,482 | 1.3E-03 | 0.49 | 59,521 | 9.9E-04 | 0.55 |
| 2nd set | 1st set | 17,771 | 4.1E-04 | 0.70 | 30,952 | 9.3E-04 | 0.56 | 41,898 | 1.8E-04 | 0.80 | 51,217 | 1.9E-07 | 0.99 | 59,375 | 1.3E-07 | 0.99 |

*P*_t_: *P* threshold, R^2^: Nagelkerke’s R^2^
